# Environmental applications of silver nanoparticles: state-of-the-art review and emerging trends

**DOI:** 10.3762/bjnano.17.49

**Published:** 2026-05-26

**Authors:** Soni Prajapati, Akash Kumar, Ranjana Singh

**Affiliations:** 1 Department of Biochemistry, King George’s Medical University, Lucknow, India; 2 Academy of Scientific and Innovative Research, Ghaziabad, India

**Keywords:** AgNPs, air application, environmental pollutants, soil applications, synthesis, water application

## Abstract

Silver nanoparticles (AgNPs) possess inherent catalytic, antimicrobial, and optical properties, making them a strong candidate for environmental applications in water, air, and soil. Indeed, various reviews are available, though a significant gap persists in addressing all environmental pollutants. This review comprehensively and critically analyses the advancement in AgNP research spanning from synthesis and characterisation to practical deployment and ecotoxicological assessment. The AgNP-based systems are evaluated regarding antimicrobial disinfection, adsorptive and catalytic/photocatalytic removal of persistent organic pollutants, and integration into antifouling nanofiltration and ultrafiltration membrane technologies used for management of water pollutants. In addition, AgNPs-assisted nanosystems in fibrous filter membranes and photocatalytic composite coatings for the removal of volatile organic compounds, particulate matter, and gaseous pollutants are reviewed. Furthermore, AgNP applications for heavy metal immobilisation, organic pollutant degradation, plant disease management, and growth promotion are assessed alongside their ecotoxicological implications. Besides remediation, environmental monitoring capabilities of AgNP-based sensing platforms are systematically reviewed across five transduction modalities, including colourimetric/UV–vis LSPR, SERS, electrochemical, fluorometric, and gas sensing, covering a broad range of analytes considered as environmental pollutants. Key challenges, including nanoparticle aggregation, long-term colloidal instability, synthesis irreproducibility, ecotoxicological risks arising from Ag^+^ ion release and environmental persistence, and the current absence of harmonised regulatory frameworks for AgNP deployment, are critically discussed. This review provides a structured, evidence-based foundation for researchers and engineers working toward the responsible, scalable application of AgNP-based technologies to address contemporary environmental challenges.

## Review

### 1 Introduction

**Figure 1:**
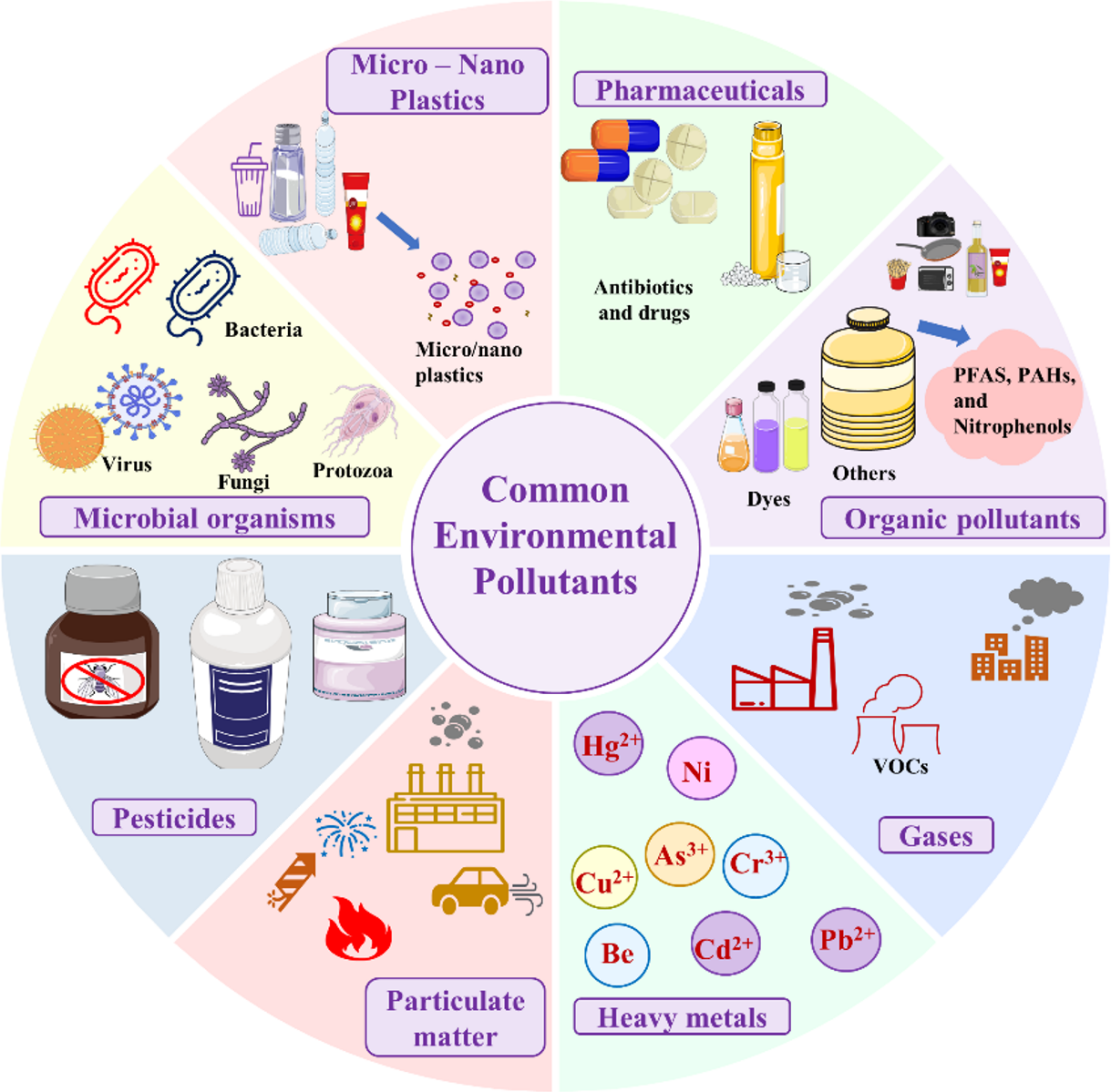
Schematic illustration of prevalent environmental contaminants affecting air, soil, and water.

Environmental pollution has intensified globally due to rapid industrialisation, technological advancements, urbanisation, and unsustainable agricultural practices. This causes significant concern and disrupts ecosystems and food cycles, ultimately affecting living beings. Anthropogenic activities generate all environmental contaminants, and industries, medical and research institutions, household waste, and agricultural practices are major contributors [1]. Pollution arises from any chemical, biological, physical, or radiological substance that impacts soil, water, air, or living beings. These include metals (e.g., lead, mercury, arsenic, cadmium, copper), particulate matter (PM2.5 and PM10), pesticides (organochlorines and phosphates), micro- and nanoplastics, antibiotics, gases, organic compounds (e.g., dyes or nitrophenols), and microorganisms [2–5]. The concentration of these pollutants in the environment is proportional to the world population and their demand for advanced technology, healthcare, and household products. Therefore, monitoring and remediation of these contaminants from the environment is the need of the hour, with cost-effective, sensitive, and selective strategies or systems foremost. Indeed, various high-throughput techniques, as well as chemical and biological methods, are available to address these pollutants, but their limited efficiency, cost-effectiveness, on-site usability, technical skills, and environmental compatibility restrict their use to specific conditions [6]. Thus, researchers have sought alternatives that maintain cost-effectiveness and a sustainable approach to managing environmental pollutants in air, water, and soil. The list of conventional and emerging environmental pollutants is represented in Figure 1.

The advent of nanoscience is a promising and effective solution for addressing all aspects of the environment (i.e., water, air, and soil). Among the broad family of engineered nanomaterials evaluated for environmental applications, silver nanoparticles (AgNPs) have attracted exceptional attention owing to their unique combination of properties that directly address the requirements of environmental monitoring and remediation. For example, the optical properties of AgNPs are primarily governed by localised surface plasmon resonance (LSPR ), which occurs in the 380–450 nm region and depends on the nanoparticles’ size and shape. The unique optical properties of nanosilver were utilised to develop LSPR-based nanosensors that detect different metal ions [7]. A surface coating of ʟ-carnosine on nanosilver enables size tuning and the detection of metal ions such as As^3+^, Cr^3+^, Cd^2+^, and Pb^2+^, while cetyltrimethylammonium bromide (CTAB) coating provides selectivity against Hg^2+^ and Cu^2+^ [8–9]. In addition, CTAB- and ʟ-carnosine-capped AgNPs exhibit catalytic activity in the removal of *p*-nitrophenol [8–9]. According to the Occupational Safety and Health Administration (OSHA; Permissible Exposure Limits, 29 CFR 1910.1000), arsenic, chromium, cadmium, lead, beryllium, and mercury are the most hazardous metal ions that can cause irreparable health impacts. These occupational thresholds are complemented by environmental exposure standards, including the US EPA National Primary Drinking Water Regulations (e.g., Pb: 0.015 mg/L; As: 0.010 mg/L) and WHO Drinking Water Quality Guidelines, which collectively underscore the necessity for ultrasensitive environmental monitoring tools capable of detecting these ions at trace concentrations in water, air, and soil matrices. Furthermore, nanosilver detected antibiotics, microplastics, bisphenol A, pesticides, bacterial pathogens, per- and polyfluoroalkyl substances (PFASs), and polycyclic aromatic hydrocarbons (PAHs) in environmental samples [10–17]. This indicates that nanosilver acts as a potent nanosensor for detecting various contaminants via different approaches, namely Raman spectroscopy, electrochemistry, colourimetry, and fluorometry [18–21]. In addition, the enhanced antimicrobial activity of AgNPs can be used for water decontamination and is widely employed in membrane filtration technology [22]. AgNPs exhibited broad-spectrum antibacterial activity, encompassing both Gram-positive and Gram-negative bacteria, as well as multidrug-resistant strains [23]. Furthermore, the catalytic activity of AgNPs was utilised to remove synthetic dyes, nitrophenol, and other organic pollutants. Methylene blue (MB), Congo red (CR), 4-nitrophenol, and 4-nitroaniline were degraded into harmless products using AgNPs stabilized by *Cestrum nocturnum* L. [24]. In a study, chitosan-based poly(chitosan-*N*-isopropylmethacrylamide-acrylic acid) microgels were used to fabricate AgNPs via borohydride reduction. The study demonstrated the catalytic removal of MB, CR, brilliant blue, rhodamine B (RhB), and methyl orange (MO) with pseudo-first-order reaction kinetics [25]. The addition of AgNPs to a matrix may improve their efficiency in removing various contaminants via adsorption mechanisms, which can be ionic or covalent, depending on the nanocomposite surface chemistry [26]. In a study, Ag-decorated reduced graphene oxide was used as an adsorbent material for removing Nile blue dye, with 94% of the dye being removed within 60 min [27]. Caravaca et al. used magnetic nanoparticles coated with AgNPs for amoxicillin removal from water, where 100% removal was achieved at different nanocomposite concentrations [28]. The removal of PFAS was achieved by formulating nanosilver with activated carbon, where perfluorooctanesulfonate (PFOS) and perfluorooctanoate (PFOA) were adsorbed at concentrations of, respectively, 454.1 and 321.2 mg/g, respectively [29]. *Echinochloa pyramidalis*-stabilized AgNPs were synthesized for the removal of PAHs with 100% efficiency [30].

AgNPs and AgNP-based nanocomposites provide an efficient system for pollutant removal, with good reusability, as evident from the above studies. However, despite their benefits, the escalating production and utilisation of AgNPs also raise concerns about their safety and the risks associated with environmental applications, in particular regarding the cytotoxicity of Ag^+^ ions [31]. The mechanisms underlying the toxicity of AgNPs towards various organisms, including humans, must be elucidated to inform risk assessment and mitigation strategies [32]. The AgNPs exhibit toxicity towards aquatic organisms, including bacteria, algae, invertebrates, and fish, with reported adverse effects such as growth inhibition, reproductive impairment, and mortality [32–33]. Moreover, various factors influence the fate and transport of AgNPs in environmental samples, including particle size, surface composition, state stability, and organic matter, which ultimately generate bioavailability and toxicity concerns [34]. The regulatory framework governing AgNP environmental applications remains fragmented across jurisdictions; while OECD, ISO, EU, and US EPA guidelines address nanoparticle characterisation and safety assessment, specific maximum contaminant levels or environmental quality standards for engineered AgNPs are largely absent.

Several reviews have addressed individual aspects of the environmental use of AgNPs, including antimicrobial applications, heavy-metal sensing, and photocatalytic degradation, in isolation. However, no single review has provided an integrated cross-domain treatment spanning sensing, photocatalysis, adsorption, membrane filtration, disinfection, and soil/agricultural applications within a unified mechanistic framework. The present review addresses most of the pollutants (emerging and conventional) by providing a comprehensive assessment of AgNPs and AgNP-based nanocomposites in environmental applications. It incorporates emerging topics including green synthesis reproducibility, nanozyme activity, the environmental fate of silver, and metrological validation of sensing platforms. It also identifies critical standardisation gaps and provides a balanced assessment of practical feasibility relative to regulatory thresholds. Together, these elements define the novelty of this review and distinguish it from prior works in the field.

### 2 Preparation and characterisation of AgNPs

**Figure 2:**
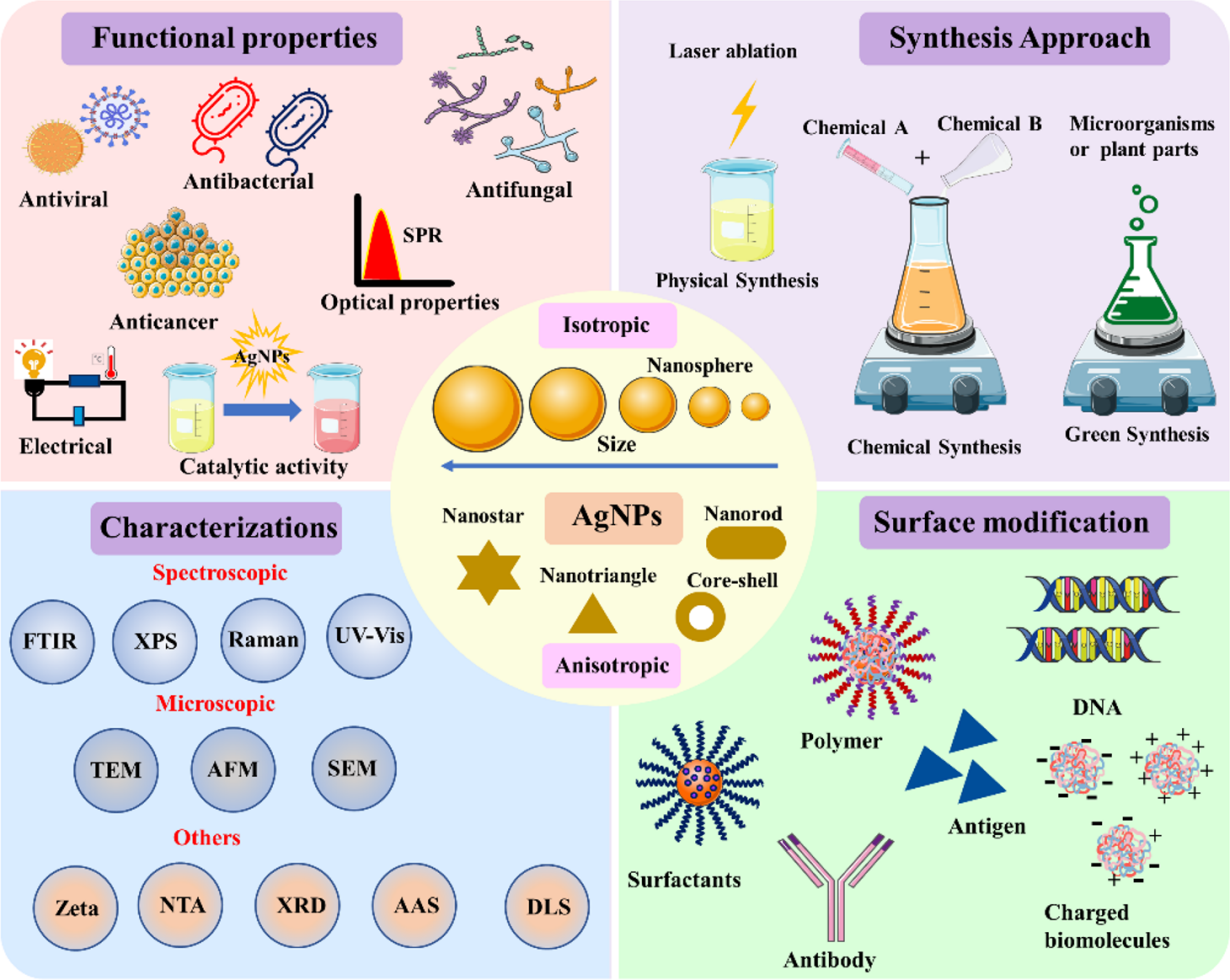
Systematic illustration of silver nanoparticle preparation methods, surface modification techniques, analytical characterization, and functional properties.

The environmental application of AgNPs is primarily determined by their functional properties, which are highly dependent on their synthesis and surface functionalization. In addition, the physicochemical properties of differently synthesised AgNPs are characterised using standard techniques before their practical use. Furthermore, surface modification with a suitable ligand or surface capping enables targeted interaction with the analyte via the exposed functional group, thus enabling selective detection. Surface capping enhances the stability of nanoparticles and their functional properties for catalytic/photocatalytic applications (Figure 2). Indeed, differently capped nanoparticles were prepared using suitable preparation methods.

#### 2.1 Preparation methods

The environmental applications of AgNPs are primarily governed by physicochemical parameters obtained through various synthetic approaches, including physical, chemical, and biological methods [35]. Each offers distinct advantages and limitations regarding particle size control, morphology, stability, and cost-effectiveness [35]. Chemical methods are commonly employed to synthesise AgNPs due to their simplicity, scalability, versatility, and ability to control size [36]. Chemical reduction methods are widely used to synthesise AgNPs, utilising reducing agents such as sodium borohydride, citrate, and ascorbic acid to reduce silver ions in solution, followed by the use of capping agents or stabilisers to prevent aggregation [36]. The capping agents can be polymers, surfactants, and small and large biomolecules, providing colloidal nanoregime stability [37]. The size-controlled synthesis of AgNPs capped with ʟ-carnosine was developed for metal ion detection and nitrophenol removal [8]. Another study highlighted capping agent-mediated size-controlled synthesis of nanoparticles for peroxide detection and antimicrobial activity [38]. PVA-AgNPs were the smallest and showed enhanced antibacterial activity and H_2_O_2_ sensing [38]. In addition, incorporating linker molecules enables selective and sensitive sensing, allowing for a design tailored to a specific application. For example, PVP-capped AgNPs were used to selectively detect Hg^2+^ in the presence of methionine as a linker molecule [39]. Laser ablation, evaporation–condensation, and sputtering fall under physical syntheses and offer the advantage of producing highly pure materials without the use of chemical reagents. However, the techniques lack monodispersed nanoparticle distributions [40].

Green methods employing plant and their parts extracts, microorganisms, or biopolymers as reducing and capping agents have gained increasing attention due to their environmental friendliness and biocompatibility [41–42]. Green synthesis can be considered an alternative to chemical and physical methods, utilising renewable resources and mild reaction conditions to produce AgNPs with tailored properties [43]. Developing new AgNPs via green synthesis has emerged as a rapid, practical approach, yielding fewer toxic nanoparticles with specific properties [44]. Plant extracts contain diverse bioactive compounds, including flavonoids, alkaloids, and polysaccharides, which act as reducing and stabilising agents in the synthesis of AgNPs [45]. It was observed that *Acacia raddiana*-stabilised AgNPs simultaneously detected Co^2+^, Hg^2+^, Pb^2+^, and Cu^2+^, indicating their role as a multimetal sensor for environmental monitoring [41]. The catalytic potential of *Plantago ovata* leaf extract-stabilised AgNPs was assessed by measuring MB and CR removal, with reaction rates of 0.056 and 0.166 min^−1^, respectively. In addition, the antifungal activity against *A. alternata* and *F. oxysporum* indicates it as a decontaminating agent [46]. The antimicrobial properties of nanosilver are highly dependent on size, shape, and surface functionalization [47]. A prior characterisation using standard techniques is necessary to understand the physicochemical properties of AgNP [48].

#### 2.2 Characterisation techniques

AgNPs are characterised to understand their physical and chemical properties, which are mainly governed by size, shape, and surface complexity. This understanding was needed before engineering AgNPs for particular applications. AgNPs are characterised using optical spectroscopy, electron microscopy, X-ray diffraction, and dynamic light scattering (DLS) to measure plasmonic absorbance, size, shape, structure, and stability [49]. Optical spectroscopy is a rapid and convenient technique for monitoring the formation of AgNPs via size- and shape-dependent absorption bands arising from LSPR upon light excitation [50]. This study also highlighted that small spherical AgNPs were highly antibacterial against *Pseudomonas aeruginosa* and *Escherichia coli* compared to larger and triangular particles [50]. Optical methods are employed to selectively detect metal ions, antibiotics, and pesticides using AgNPs, thereby facilitating environmental monitoring [19]. Other techniques include DLS, which works on the principle of Brownian motion and measures a particle's hydrodynamic size and surface charge in a colloidal solution [49]. DLS measures the size distribution and stability of AgNPs in solution, providing valuable information on their tendency to aggregate or agglomerate [51]. DLS measurements are suitable for isotropic particles and help elucidate the surface properties of AgNPs through their interactions with the surrounding medium. Zeta potential measurements are used to assess the surface charge and stability of silver nanoparticles; higher absolute values (>±30 mV) indicate better colloidal stability and resistance to aggregation [52]. This surface charge also validated the surface functionalization with molecules of interest. However, two separate cuvettes were used for the measurement, and the absorption coefficient (*k* = 3.99) and refractive index (*n* = 0.135) of AgNPs need to be added. In addition, a similar principle was employed by the nanoparticle tracking analyser, which calculated the AgNP concentration as particles per millilitre [53]. Microscopic techniques, such as transmission electron microscopy, provide high-resolution images of individual nanoparticles, confirming morphology, size distribution, and elemental composition when coupled with energy-dispersive X-ray analysis [49]. TEM analysis is performed on copper grids with varying mesh sizes and coatings, with carbon/formvar-coated copper grids commonly preferred. Scanning electron microscopy (SEM) provides information on the silver nanoparticles’ surface properties and the aggregation state of dried nanoparticles using carbon or silicon wafers as the primary substrate [49]. The X-ray diffraction pattern reveal that AgNP crystal growth occurs at different facets depending on the NPs [49]. The most common facets include 111, 200, 220, and 311, corresponding to 2θ angles of 38.2°, 44.4°, 64.6°, and 77.5°, respectively [54]. Using the Scherrer equation, these 2θ values yield the nanoparticle’s crystallite size [54]. Furthermore, Fourier-transform infrared (FTIR) spectroscopy provides evidence of successful surface capping or functionalization of the desired molecules on nanoparticles [49]. Surface-enhanced Raman spectroscopy (SERS) using AgNP nanocomposites could selectively detect polystyrene nanoparticles with a detection limit of 14 μg/mL [55]. X-ray photoelectron spectroscopy (XPS) provides information on the elemental composition, surface oxidation states, and electronic configuration of AgNPs [56]. This technique can detect environmental contaminants using AgNPs as the probe. The ICPMS (Inductively Coupled Plasma Mass Spectrometry) and AAS (Atomic Absorption Spectroscopy ) were used to measure unknown metal (Ag) content by plotting linear curves using the known Ag standard [49]. Electrochemical techniques, including voltammetry, potentiometry, and amperometry, were used to monitor metal ions, pharmaceuticals, endocrine disruptors, ammonia, and phenolic compounds using AgNPs [18]. Fluorescence spectroscopy provides information on fluorescence-based detection of environmental pollutants via fluorescence enhancement or quenching mechanisms. A study reported that carbon dot/AgNP-based nanosystems selectively detected Hg^2+^ ions with a detection limit of 2.22 × 10^−8^ M via a turn-on mechanism [57]. In addition, the environmental application of AgNPs can be affected by various factors, such as surface composition, size, temperature, pH, concentration, and shape.

#### 2.3 Factors affecting AgNP properties relevant to environmental applications

Nanosilver and its properties are well-suited for various ecological applications and can be engineered by varying synthesis parameters that affect AgNP properties. The application of AgNPs depends on factors such as size/shape, capping, pH, temperature, and NP concentration, which can be optimised through the synthesis approach. The size and shape of AgNPs significantly influence their properties and applications, with smaller particles exhibiting enhanced functional properties due to their higher surface-area-to-volume ratio [58]. A study reported that luteolin tetraphosphate-stabilised nanosilver exhibited size- and shape-dependent antimicrobial activity [59]. The study concluded that 100% bacterial and fungal growth was inhibited, indicating potential in water and air filtration systems [59]. Another study highlighted the role of AgNP size and shape in catalytic MB removal [60]. AgNPs with controlled size of 10 nm and nanocapsular shape showed selective detection of Cu^2+^ ions with LOD of 2.6 × 10^−9^ mol/L [61]. Size-tuned AgNPs (1–100 nm) were synthesised using trisodium citrate as a capping agent to assess the impact of nanoparticle size on bacterial strain [62]. The study found that 5 nm AgNPs have a greater effect on killing bacterial strains compared to other sizes [62]. Collectively, these studies indicate that morphology and size significantly influence AgNP performance; however, direct cross-study comparison remains challenging owing to differences in synthesis routes, capping agents, test matrices, and reporting conventions. While general trends (e.g., smaller spherical AgNPs typically exhibiting stronger antimicrobial activity and greater LSPR sensitivity) are well-supported, the magnitude of these effects is system-specific and should not be stated categorically without accounting for confounding experimental variables. Besides size and shape, capping/stabilising agents prevent aggregation and provide colloidal stability to AgNPs in the surrounding medium. Selecting the appropriate capping agent from polymers, surfactants, biomolecules, and green extracts is essential to ensure long-term stability and prevent agglomeration, thereby making it suitable for environmental applications [63]. In addition, surface functionalization or coating with biocompatible polymers or targeting ligands can enhance stability, dispersibility, and biocompatibility for specific ecological applications [64]. For example, differently capped AgNPs (e.g., PEG, EDTA, PVP, or PVA) exhibited NP size variations, ultimately affecting antimicrobial and sensing applications. PVA provided the smallest NP size, yielding enhanced antibacterial activity and H_2_O_2_ sensing abilities [38]. In another study, *Acacia lignin*-AgNPs showed catalytic, sensing, and antimicrobial activity [65]. The results confirmed that MB and *p*-nitrophenol were catalytically removed at rates of 0.00098 and 0.01525 min^−1^, respectively. Additionally, NPs demonstrated metal ion sensing and antibacterial activity against various strains [65]. The above studies concluded that the capping agent played a prominent role in nanoparticle-mediated applications by providing an interaction site for targeted molecules and by stabilising NPs. The pH-dependent detection of metal ions using PVP-AgNPs was reported, with Pb^2+^ detected at pH 9.6 at a LOD of 14.4 nM [66]. The reaction pH influences stability and dissolution of the AgNPs, which are among the main factors in AgNPs-mediated applications [67]. Particle aggregation is a critical factor influencing the behaviour and toxicity of AgNPs in ecological systems [64]. Surface charge affects the electrostatic interactions between nanoparticles and other charged species in the environment, influencing their aggregation behaviour and mobility. The surface chemistry of silver nanoparticles, including surface coatings or functional groups, can alter their reactivity, stability, and interactions with pollutants and microorganisms [68]. Environmental standardization and stabilization of AgNPs surface charge are essential in biological systems for studying interactions [69]. Response surface methodology is also helpful in optimizing experimental parameters and AgNP preparation by minimising the number of trials and errors [70].

#### 3 Environmental application of silver nanoparticles

**Figure 3:**
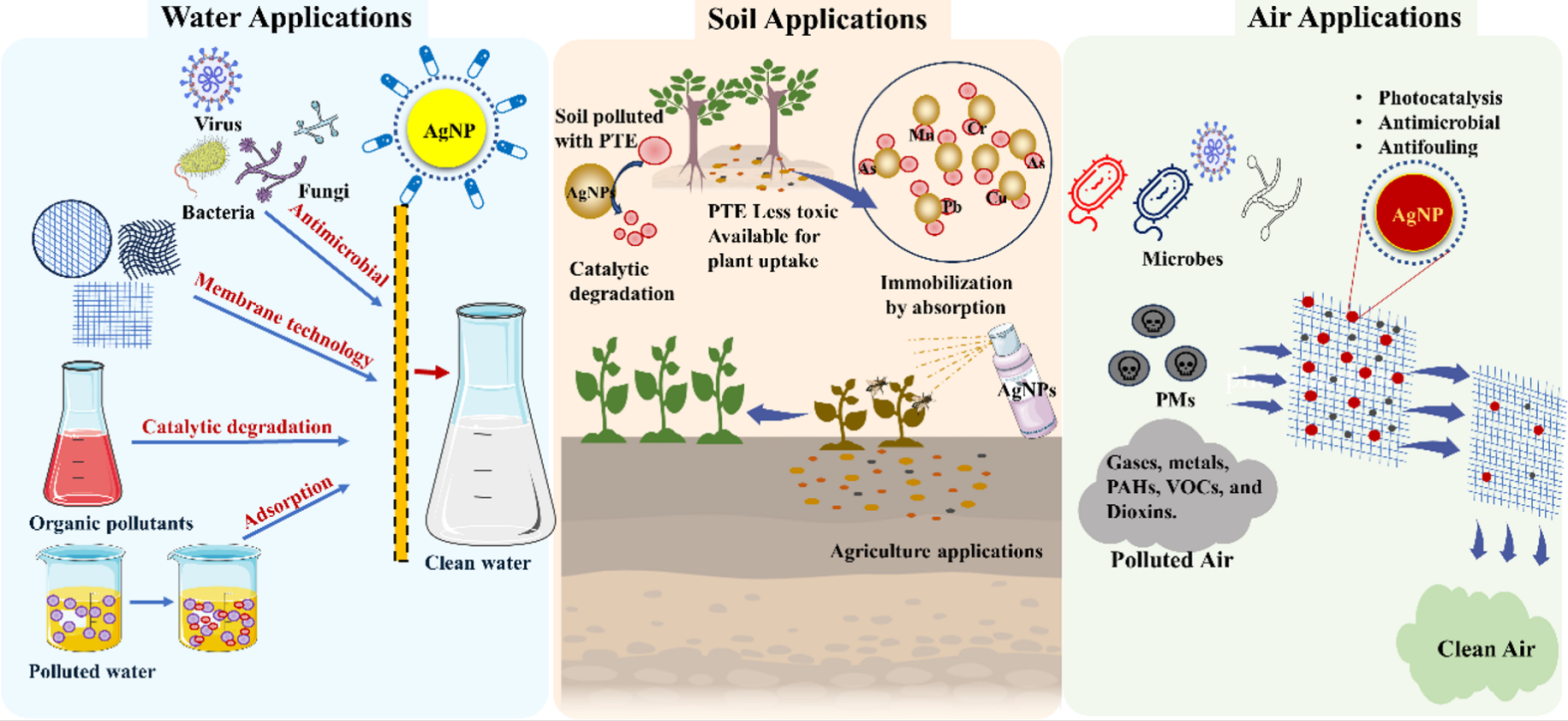
Application of silver nanoparticles in the management of air, water and soil pollutants.

Silver nanoparticles possess inherent properties that can be utilised for air, water and soil applications, as shown in Figure 3.

#### 3.1 Water applications

The global demand for clean and safe drinking water continues to increase due to population growth, urbanisation, and industrialisation. Clean water and sanitation are part of Sustainable Development Goal 6 (SDG6), which must be achieved by 2030. Nanosilver has emerged as a promising material for water treatment, offering efficient and cost-effective solutions for removing, monitoring, and disinfecting water sources [71]. Nanosilver’s catalytic and antimicrobial properties make it a highly efficient material in treating water pollutants.

**3.1.1 Antimicrobial properties and disinfection.** Nanosilver showed broad-spectrum antimicrobial activity (bacteria, viruses, and fungi) and can serve as an effective disinfectant for water applications [7,72]. Various mechanisms, including disruption of cellular membranes, interference with DNA replication, and the generation of reactive oxygen species (ROS), mediate the antimicrobial effect [73]. AgNPs stabilised with *Hormophysa triquetra* extract inhibited *Escherichia coli*, *Bacillus subtilis*, *Staphylococcus aureus*, *Pseudomonas stutzeri*, and *Pseudomonas fragi* in a solvent-dependent manner [74]. AgNPs showed an inhibition zone of 22.5 and 25 mm in chloroform and ethanol, respectively, against *P. fragi*. In contrast, inhibition of *E. coli* was achieved in methanol (23.5 mm) [74]. In another study, AgNPs stabilized with *Eriobotrya japonica* seed extract showed enhanced antibacterial activity compared to penicillin. The author found that *E. coli*, *Klebsiella*, and *S. mutans* showed inhibition zones of 20 and 15 mm upon exposure to 150 mg/mL AgNPs [75]. In addition, AgNPs demonstrated efficacy in inactivating viruses, including adenovirus, hepatitis B, herpes simplex, influenza A, Chikungunya virus, and Zika virus, by disrupting their protein coats and interfering with their replication mechanisms [76]. Waterborne viral infections include adenovirus, astrovirus, hepatitis A and E viruses, rotavirus, and norovirus [77]. Additionally, human urine excretion can contain polyomaviruses and cytomegalovirus, which can contaminate water and spread through water sources [77]. Nanosilver can kill viruses and make water safe to drink under controlled laboratory conditions, but translating this to potable water treatment would require comprehensive validation in realistic matrices, assessment of Ag^+^ release, and long-term safety evaluation. Nanosilver coated with tannic acid significantly inhibits adenovirus type 3 (Ad3) in HeLa cells, serving as a model system [78]. The inhibition mechanism involves structural and DNA damage to Ad3 [78]. Another study assessed the dose- and size-dependent antiviral effects of AgNPs against feline calicivirus, which shares a similar genetic makeup with human norovirus [79]. The study found that 10 nm AgNPs significantly reduced viral load (6.5 log10 viral titer) at concentrations of 50 and 100 μg/mL. The protein expression analysis confirmed that the capsid protein showed a reduction in signal, indicating that AgNPs with smaller size-selectivity interact with the virus capsid [79]. Furthermore, nanosilver exhibits antifungal properties against waterborne fungi, including *Acremonium*, *Alternaria*, *Aspergillus*, *Chaetomium*, *Fusarium*, *Mucor*, *Lichtheimia*, *Paecilomyces*, *Penicillium*, *Phoma*, *Scopulariopsis*, and *Trichoderma* [80]. Nanosilver stabilised with an extract of *Bacillus thuringiensis* MAE 6 was assessed for its antifungal activity against four *Aspergillus* strains (*A. niger*, *A. flavus*, *A. terreus*, and *A. fumigatus*) at 500 μg/mL [81]. The inhibition zone and MIC values were 16, 26, 20, and 19 mm, and 125, 15.62, 62.5, and 62.5 μg/mL, respectively [81]. Another concern are waterborne protozoa, which have a significant impact on global health. A recent review suggested that *Giardia* and *Cryptosporidium* are prominent, whereas others include *Cyclospora cayetanensis*, *Dientamoeba fragilis*, *Toxoplasma gondii*, *Blastocystis hominis*, *Entamoeba histolytica*, and *Microsporidia* or *Naegleria fowleri* [82]. A study indicated that commercial AgNPs showed anti-Giardia activity in combination with metronidazole (an antiparasitic), but the efficacy was not optimal [83]. Another study using stabilized AgNPs from *Astragalus ecbatanus* treated *Giardia lamblia* infection in vitro and in vivo model systems [84]. The study found that AgNPs killed cysts at 200 and 300 μg/mL after 4 and 2 h, respectively, whereas trophozoite reduction was observed at 100, 200, and 300 μg/mL after 4, 2, and 1 h, respectively [84]. The above studies, along with those in Table 1, confirmed the potential of AgNPs as disinfectants for water treatment applications. However, the environmental remediation perspective of AgNPs needs to be discussed to assess their full potential.

**Table 1:** AgNPs explored as antimicrobial agents, including nanomaterials, tested microorganisms, limitations, and outcomes.

Nanomaterial	Tested microorganism and dose	Outcomes	Limitations	Ref.

*Lysiloma acapulcensis* extract AgNPs	*E. coli*, *S. aureus*, *P. aeruginosa*, *C. albicans*; dose: 0.06–5 µg/mL	green one-pot synthesis (≈5 nm, spherical); significantly higher potency than chemically synthesized AgNPs; broad-spectrum, including fungi; low cytotoxicity; 0.06–0.13 (MIC)/0.13–0.25 (MBC); ZOI: 18.0 ± 1.3, 16.0 ± 1,(15.0 ± 0.5), (18.0 ± 1.3)	activity depends on the phytochemical profile of the plant (batch variability); long-term stability not reported	[85]
Pu-erh tea leaf extract AgNPs	*E. coli*, *K. pneumoniae*, *S. Typhimurium*, *S. Enteritidis*; dose: 3.9–7.8 µg/mL	ultrasmall size (4.06 nm, spherical) gives rapid bactericidal action (>99.9% kill in 1–2 h); effective against foodborne Gram-negative pathogens; sustainable synthesis; 3.9–7.8 (MIC)/3.9–7.8 (MBC)	slightly higher MIC for *E. coli*; no long-term reusability data; no batch variation assessment	[86]
*Exiguobacterium aurantiacum*-AgNPs	*S. aureus*	biogenic synthesis; (spherical, 121.44 nm) antibacterial, dye remediation and anticancer potential; 2–32 (MIC)/32–150 (MBC); ZOI: 17.5	activity reported only for *S. aureus*; no cytotoxicity data for antimicrobial concentrations	[87]
Urea gemini surfactant-capped AgNPs	*S. aureus*, *E.coli*, *C. albicans*; dose: at *n*_Ag_/*n*_surf_ = 2.5–10	structure-tunable activity via spacer length; high colloidal stability (+41–+64 mV zeta); synergistic Ag + surfactant effect; cubic shape for some spacers; size 50–150 nm	larger particle size reduces potency vs <20 nm AgNPs; activity weaker against yeast; no zone of inhibition data; full MIC numbers graphical only	[88]
PVP-capped AgNPs	*S. aureus*, *E. coli*; tested as aqueous suspensions (initial Ag precursor 1–5 mM); PVP capping at 1–5 wt %	environmentally benign synthesis (17–23 nm, iso-anisotropic); tunable antimicrobial via PVP MW and concentration; excellent 6-month stability; anisotropic particles (PVP 360 K) enhance activity	higher PVP concentration reduces Ag^+^ leaching (smaller zones); MW-dependent differential effect; no MIC/MBC or exact zone diameters provided (qualitative only)	[89]
PVP-stabilised AgNPs	*P. aeruginosa* (*P.a*), *S. aureus* (*S.a*); dose standardised to deliver ≈400 ppm soluble Ag^+^ (0.3 mL of 1 mg/mL suspension)	notable broad-spectrum antibacterial and anti-virulence activity (81.87% viability reduction *P. aeruginosa*, 59.4% *S. aureus*; 80.2% metabolic inhibition *P.a*, 58.4% *S.a*; strong biofilm/pigment/enzyme/motility inhibition; DNA/protein/K^+^ leakage); stable aqueous suspension; comparable or superior to AgNO_3_ in most assays; mean size 28.9 ± 7 nm (spherical)	less potent than electrolysed Ag^+^; species-specific susceptibility (*P.a* more affected than *S.a*); moderate colloidal stability (zeta −14.4 mV); no MIC/MBC or zone data; no direct comparison of Ag^+^ leaching	[90]
*Azadirachta indica* and *Justicia adhatoda* leaf extract AgNPs	*E. coli*, *B. subtilis*	eco-friendly, cost-effective green synthesis using common medicinal plants (cryatallite size 2.2 and 9.0 nm, spherical to anisotropic); strong broad-spectrum activity; optimal activity at medium extract concentrations; crystalline FCC structure confirmed	no exact MIC/MBC; concentration-dependent (lower/higher conc. less effective); no stability assessment, and Ag leaching	[91]
Bergamot pomace extract AgNPs	*Pseudomonas syringae* pv. tomato, *Xanthomonas campestris* pv. Vesicatoria; dose 5–50 µg/mL	eco-friendly synthesis from agricultural waste; strong dose-dependent antibacterial (50% inhibition at 5 µg/mL vs *P. syringae*, 90% vs *X. campestris*; complete inhibition at 20–50 µg/mL); outperforms AgNO_3_ against *X. campestris*; dual phytostimulatory + antimicrobial activity; mean size 15–20 nm (spherical)	no zone of inhibition or MIC/MBC values; liquid-culture assay only (no disk diffusion); species-specific sensitivity; full quantitative data limited to % growth inhibition	[92]
Citrus-peel-derived AgNPs (lemon L-AgNPs and tangerine T-AgNPs)	*S. aureus*, *S. typhimurium*, *L. monocytogenes*; incorporated in alginate-gelatin films at 20% (w/w); or 30 μL suspension on discs for testing	green synthesis from citrus peel waste (size: L-AgNPs 28.1 ± 17 nm and, T-AgNPs 25.2 ± 10 nm, spherical to aggregation); broad-spectrum activity; superior for L-AgNPs vs T-AgNPs; strong antimicrobial in films + antioxidant effect; inhibition zone: L-AgNPs: 13.50–16.50 mm; T-AgNPs: 9.75–14.10 mm	no direct Ag^+^ leaching comparison; MIC/MBC values; zone data only for incorporated films; T-AgNPs weaker than L-AgNPs	[93]
In situ biogenic AgNPs on commercial sponge matrix	*E. coli*, *S. aureus*, *C. albicans*	eco-friendly synthesis (size 54 ± 14 nm, spherical) on porous sponge; >99.99999% inactivation for bacteria and yeast; excellent filtration removal (up to 6.2–6.4 log CFU/mL over 6 cycles); retains activity after 5000 cm abrasion and 400 bending cycles	no zone of inhibition, MIC, or MBC values; activity measured as log reduction only; performance specific to surface contact/filtration; no Ag^+^ leaching quantification	[94]
Fruit peel extract AgNPs	*B. cereus*, *S. aureus*, *E. coli*, *M. morganii*; *A. niger*, *A. alternata*, *P. digitatum*, *F. oxysporum*; tested at 10–40 µg/mL and stock 1 mg/mL	green synthesis from abundant fruit-peel agricultural waste (banana (56.09 nm, spherical) + orange, 53.44 nm, spherical); broad-spectrum activity against human + plant pathogens; very low MIC values (3.125–12.5 µg/mL); concentration-dependent enhancement; inhibition: BPAgNPs 6.67–22.3 mm; OPAgNPs 7.33–24.67 mm	no MBC values; no Ag^+^ leaching or long-term stability data; tested only on fruit-isolated strains (not standard ATCC panels); no filtration/platform application shown	[95]

**3.1.2 Pollutant removal or degradation.** The antimicrobial action of AgNPs has been explored for water treatment and purification. Despite this, several pollutants, including metal ions, pesticides, and dyes, are present in water and significantly contribute to water pollution. To obtain purified water, these pollutants must be removed from water sources through adsorption/absorption or catalytic degradation into harmless molecules.

**3.1.2.1 Removal via adsorption/absorption.** Nanosilver or nanocomposites containing AgNPs are predominantly used for pollutant removal via ionic or covalent interactions. The colloidal nanosilver has a limited adsorption capacity, which was increased by adding other materials. This includes carbon-based nanocomposites (activated carbon, graphene, and nanotubes), polymer-based nanocomposites (chitosan and alginate), and metal oxide-based nanocomposites (iron, zinc, and titanium) [26]. A recent study confirmed the removal of three metals (Zn^2+^, Pb^2+^, and Fe^3+^) from the aqueous phase using AgNPs immobilised on banana leaf powder [96]. The study reported that pH-dependent metal adsorption capacities (190, 244, and 288 mg/g) and removal efficiencies (79, 88, and 91%) were achieved for Zn^2+^, Pb^2+^, and Fe^3+^ within 40 min of contact [96]. Another study used Murcott mandarin-stabilised AgNPs to remove Pb^2+^ ions, achieving an adsorption of 42.7 mg/g in 60 min at pH 5.5 [97]. Similar to metal ions, dye removal was achieved using *Shorea robusta* leaf biochar-stabilised AgNPs, yielding more than 90% removal of RhB and CR [98]. The mechanism underlying removal was surface complexation via electrostatic interactions in both dyes, with hydrogen bonding occurring only in CR [98]. MB removal was achieved using AgNPs mediated by *Salvinia molesta*. The maximum adsorption of 121.04 mg/g was obtained at pH 4 and 35 °C. Additionally, the nanosilver composite exhibited an enhanced adsorption capacity against PFAS, an emerging environmental contaminant. The two nanocomposites were prepared using chemically and physically activated carbon from maize tassel [29]. Chemically activated nanocomposites exhibited the highest adsorption capacities for PFOS and PFOA, at 454.1 mg/g and 321.2 mg/g, respectively [29]. It was also observed that nanosilver interacted with microplastics in water and adsorbed onto their surfaces, including polypropylene, polyethene, and polystyrene [99]. However, the removal of microplastics from the nanosilver composite is still inconclusive. Despite that, nanosilver possesses functional properties against various environmental contaminants. A nanocomposite combined with magnetic nanoparticles removed amoxicillin (a common antibiotic) from water sources with 100% efficiency [28]. Dose-dependent removal of the antibiotic (i.e., 10 and 100 mg/L) was achieved with 100 and 500 μL of the nanocomposite at neutral pH within 15 min. The nanocomposite was reused up to three cycles with 93% efficiency, indicating potentail as a safe, cost-effective nanosystem for environmental remediation [28]. A nanocomposite composed of magnetic nanoparticles and AgNPs showed the removal of the drug ibuprofen from water. 93% removal was achieved at neutral pH, room temperature, and a low adsorbent dose (7 mg in 500 μL), along with reusability of up to three generations (89.3%) [100]. From the above discussion it was confirmed that nanosilver alone or in combination showed efficinency in removal of water pollutant via adsorption mechanism.

Table 2 indicates types of adsorbent, advantage, limitations and initial pollutant concentrations employed in most AgNP adsorption studies remain high (typically 10–500 mg·L^−1^) to demonstrate maximum capacity, far exceeding WHO and US EPA regulatory thresholds for drinking water (Pb: 0.015 mg·L^−1^ action level; Cd: 0.005 mg·L^−1^ MCL; As: 0.010 mg·L^−1^). This concentration gap highlights the necessity for additional validation at environmentally relevant trace levels. Finally, Ag leaching from the adsorbent must be systematically quantified and reported alongside removal data to evaluate secondary contamination risk, long-term stability, and regulatory compliance factors that remain inconsistently addressed in many studies and are essential for scaling AgNP materials beyond laboratory proof-of-concept. In addition to adsorption, nanosilver exhibits strong catalytic potential against various water pollutants.

**Table 2:** AgNP and AgNP-based nanocomposite for adsorption-assisted removal of pollutants, along with critical parameters.

Adsorbent material	Pollutant and adsorption capacity (mg/g)	Experimental parameters	kinetics (*R*^2^), time (min) and reusability (cycles)	Matrix	Limitations	Advantages	Ref.

Ag-NCs (silver nanocomposites from leaf/stem/root extracts of *Echinochloa pyramidalis)*	naphthalene, acenaphthylene, acenaphthene, fluorene, phenanthrene, anthracene, fluoranthene, pyrene, chrysene, benzo[*a*]anthracene, benzo[*b*]fluoranthene, benzo[*k*]fluoranthene, benzo[*a*]pyrene, indeno[1,2,3-*cd*]pyrene, dibenzo[*ah*]anthracene, benzo[*ghi*]perylene) in bitumen seepage water	column adsorption, contact 10–90 min, natural bitumen seepage water	NA, 90	bitumen seepage water	no *q*_max_ or isotherm/kinetics modelling; no Ag leaching quantification; performance % only; no pH/temperature/dose optimization details	sustainable green synthesis; mesoporous structure + high surface area/volume ratio; exceptional PAH removal; easy regeneration with acetone	[30]
ZIF-8/AgNPs nanocomposites	Cd^2+^ (76.34), Cu^2+^ (79.36)	nanocomposite (20 mg); Cd (II) and Cu (II) solutions (10, 30, 50, 100 mg/L). nanocomposite (0–10%, w/w), pH (4–8), temp (10–50 °C) time (1–120 min)	pseudo 2nd order (0.99), 40, 5 (≈80% retention)	tap, river, sea and wastewater	limited reusability data; electrospinning complexity; no Ag leaching assessment	high capacity for Cd/Cu; integrated antibacterial properties	[101]
Magnetically synthesised AgNPs coated with graphene oxide	Pb^2+^ (326.77), Hg^2+^ (300.37), Cd^2+^ (219.13), naphthalene (71.93), phenol (58.11), fluorene (67.77)	pH (2,7, 13); temp (298 K); initial conc. 2.5 to 20 mg/mL, regeneration (pH 5)	pseudo 2nd order (0.99), 360 (6 h), (≈78–80% for metals; ≈20% drop for organics)	refining wastewater	breakthrough in continuous flow; matrix interference; Ag leaching assessment	magnetic recovery; high multipollutant capacity; chemisorption mechanism	[102]
AgNPs/GO/chitosan nanocomposite	Fe^3+^ (301.04), Cr^6+^ (234.15)	Cr^6+^: 50 ppm, 0.1 g/100 mL, pH 4, 30 °C, 150 rpm; Fe^3+^: 40 ppm, 0.02 g/100 mL, pH 6, 30 °C, 250 rpm	pseudo 2nd order (0.98); nonlinear Dubinin–Radushkevich model, 30 (Fe^3+^), 80 (Cr^6+^), 4 (≈20% retention)	synthetic aqueous solutions, as well as real industrial wastewater	pH and temp-dependent; limited reusability; interference at high initial concentrations; Ag leaching assessment	very high *q*_max_; green synthesis; high surface area (922 m^2^/g); effective in both batch and column mode; waste-to-treasure approach	[103]
*Moringa oleifera* leaf extract-AgNPs	Pb^2+^ (123.63), Cd^2+^ (294.15), Cr^3+^ (122.93)	initial conc. 50–600 mg/L (optimized), dose 0.1 g/25 mL, pH 6 (Cd/Cr), pH 10 (Pb), temp 35–40 °C	pseudo 2nd order (0.99), 150	wastewater (synthetic and real)	pH and temp dependent, limited detailed reusability cycles; potential batch variability in green synthesis;Ag leaching assessment	green synthesis; high adsorption and fast kinetics; spontaneous/exothermic process; effective for multimetal removal	[104]
*Solanum tuberosum* peel AgNPs	bromophenol blue dye (9.6 (with NP) and 8.157 (without NP))	pH 4, initial conc. (50 mg/mL), adsorbent dose (0.06 g), temp (300–323 K), sonication (120 min)	pseudo 2nd order (0.98), 120, 5 (≈85–90% retention)	aqueous solution	low *q*_max_ compared to high-surface-area adsorbents; ultrasound energy input required; pH-sensitive; potential interference from anions/cations in real wastewater	ultrasonic assistance boosts efficiency, green synthesis, good reusability, stability, and low-cost agricultural waste valorization	[105]
AgNPs	phosphorus (177.28)	pH 6, initial conc. 10 mg/L, NP 0.233–2.23 mg, temp 25 ± 2 °C	pseudo 2nd order (0.92) and Langmuir isotherm (0.95), 120	aqueous solution	no reusability or regeneration data; potential high Ag leaching risk in bare NPs; limited matrix testing; pH-sensitive	effective batch removal; simple AgNP adsorbent; good for phosphorus from aqueous media; influences of pH, contact time, dose, and initial concentration studied	[106]
Magnetic NP-AgNPs	nitrates (357.14)	2, 5, 10 and 50 mg/L of nitrate at pH 5, RT, and 50, 100, 250 and 500 µL of nanoparticles, respectively	pseudo 2nd order (0.99), 1, 4 (regeneration after 2 cycles; 90% after 3rd and 80% after 4th cycle)	aqueous solutions	no Ag leaching estimation; efficiency decreases at very high initial concentrations or extreme pH; competition from co-ions	high removal efficiency; fast kinetics; easy magnetic separation/recovery; good reusability; effective in real contaminated waters; simple synthesis	[107]
*Ligustrum lucidum* leaf extract-mediated AgNPs and AgNPs-chitosan nano-adsorbent	methylene blue (70% removal)	pH 6, initial concentration 5 mg/L, adsorbent dosage 0.005 g, temp 30–35 °C, US power 80 W	langmuir isotherm (absorption: 0.97; sonoabsorption: 0.99), 12	aqueous wastewater	no explicit *q*_max_ value; reusability not quantitatively tested; ultrasound energy required; pH-dependent; potential interference from real wastewater ions	green synthesis; ultrasound boosts removal; chitosan modification enhances capacity/stability; dual function (adsorption + antimicrobial activity); low-cost waste-derived	[108]
*Ophiorrhiza mungos*-mediated AgNPs	methylene blue (88.1 ± 1.74% with NP, 38.25 ± 0.91% with extract)	pH 7.4, temp (298–318 K, initial concentration 10 mg/L adsorbent dose 600 mg/L), dark	pseudo 2nd order (0.99) and Langmuir isotherm, 60, 5	aqueous solution	no detailed desorption agent or % drop quantification per cycle; potential aggregation in high ionic strength real wastewater not tested	green biosynthesis; spontaneous/endothermic process; good reusable up to 5 cycles; eco-friendly, sustainable, low-cost	[109]
*Justicia schimperiana* leaf extract AgNPs	Cr^6+^ (128.70 green NP and 34.38 with citrate NP)	adsorbent dose 25 mg, pH 3, initial concentration 15–35 ppm, temp: RT	pseudo 1st order (0.99: green AgNP) and pseudo 2nd order (citrate-AgNP), 30	aqueous solution; real leather industry wastewater	no reusability tested; no Ag leaching quantification; pH-dependent; potential agglomeration during green synthesis; performance drops at higher concentrations	high adsorption capacity and efficiency; green eco-friendly synthesis; Langmuir fit; effective in real leather wastewater; high surface area and promising for Cr(VI) remediation	[110]
Chitosan–AgNP composite	atrazine (0.5 (batch) and 115 µg/mL (column)	adsorbent 0.5–2 g; pesticide (1–25 ppm); pH 7	NA, 65, 5 (≈50% retention after 5th cycle)	spiked atrazine solution in ultrapure deionized	column saturates prematurely; adsorption capacity decreases after repeated cycles; limited mechanical strength of beads; no Ag leaching estimation	simple, cheap, green point-of-use household filtration system; 98% removal for 1 ppm Atrazine; better performance than some indigenous materials (rice bran, bagasse fly ash); effective in a real environment	[111]
CAMTAg (chemically activated maize tassel silver nanocomposite-activated carbon)	PFOS (454.1, 0.91 mmol/g) and PFOA (321.2, 0.78 mmol/g)	pH 2, dosage 0.05 g, initial PFAS 0.025–0.1 mg/L, temp 25 °C, agitation 180 rpm, 24 h equilibrium	Freundlich isotherm, 1440 (24 h)	aqueous solution (spiked ultrapure water)	no reusability data; optimum pH 2 (acidic); No leaching data; no real wastewater/column tests	highest *q*_max_ among agro-waste Ag-composites; spontaneous (Δ*G*/Δ*H* negative); electrostatic + hydrophobic	[29]

**3.1.2.2 Removal via catalytic degradation.** The AgNPs possess inherent catalytic properties in the nanoregime for the degradation of various water pollutants, including dyes, nitrophenols, and pharmaceuticals. Studies have highlighted the role of AgNPs as nanocatalysts in the degradation of toxic dyes, which contribute to persistent water pollution [25]. The nanocatalytic activity of AgNPs stabilised with groundnut oil cake was assessed using orange II dye, achieving 99.3% dye degradation in 120 min [112]. This study suggested the potential of green AgNPs in water treatment applications. Another study degraded 94.57% of turquoise blue (a textile dye) at 125 mg/L using *Spondias pinnata* leaf extract stabilized AgNPs. Specific parameters, including pH 5, 6 mL of AgNPs, 150 min, and a 50 W UV lamp, were used for efficient dye degradation [113]. Fenton-like oxidation of organic dyes using a nanocomposite based on AgNPs was employed to degrade MO, MB, and RhB. The dye-degradation efficiency of the nanocomposite exceeded 99% in both individual and mixed samples after 30 and 240 min, respectively [114]. The dye-degradation mechanisms are primarily governed by the generation of ROS, which further degrade dyes, with the synergistic effect of both AgNPs and iron oxide nanoparticles [114]. AgNPs stabilized with *Terminalia arjuna* showed degradation of dyes as well as nitrophenols. High degradation rates of 86.68%, 93.60%, 92.20%, and 88.80% for MO (0.166 min^−1^), MB (0.138 min^−1^), CR (0.182 min^−1^), and 4NP (0.142 min^−1^), respectively, were achieved within 20 min [115]. This study confirmed that AgNPs can simultaneously degrade multiple dyes and nitrophenols, indicating their potential as a potent nanocatalyst in water treatment. In the list of pollutants, microplastics are emerging as a hazard to living beings. Titanium dioxide nanotubes doped with Ag degraded polyethene microplastics by up to 18% after 90 min under UV-C exposure, compared to undoped nanotubes (17.33%), indicating a synergistic effect of doping. Upon doping, Ag was observed to be dispersed on the nanotube surface, either as Ag or Ag_2_O nanoparticles [116]. Another study confirmed similar results on Ag-doped titanium dioxide, where 100% degradation of polyethene (125–150 μM) was achieved at 100 ppm in 120 min of UV exposure [117]. Microplastics, such as polyamide 66, were degraded by Ag-doped TiO_2_, with increased efficiency observed with Ag doping [118]. 1.5% Ag/TiO_2_ achieved a 58.9% removal, which significantly increased to 100% upon increasing the catalyst dose (3:1) after 4 h of UV-A exposure. The underlying mechanism includes strong light absorption, a lower bandgap energy, and the generation of UV-induced electron–hole pairs [118]. The role of persulfate-conjugated Ag^+^ in removing the carboxylic PFAS perfluorooctanoic acid was demonstrated through Fenton oxidation at 20 °C, where fluorine was released along with other products [119]. A study showed ciprofloxacin removal via photocatalysis using a nanocomposite (Ag/silica), achieving 98% degradation at pH 6.7, 180 min, and a 0.12 g of catalyst [120]. Anthracene and benzene photocatalytic degradation were achieved using Ag-, Cu-, and Ni-based nanocomposites [121]. The maximum degradation was obtained under specific conditions, including 10 μg/mL (nano-composite), 5 (pH), 2 μg/mL (anthracene and benzene), and UV radiation. Freundlich and Langmuir isotherms confirmed PAH degradation, with *R*^2^ values of 0.9894 and 0.9885 for benzene and anthracene, respectively [121]. These studies confirmed that the nanosilver’s catalytic properties are significantly utilised for water pollutant removal.

**3.1.2.2.1 Mechanism of catalysis/photocatalysis.** The catalytic and photocatalytic activity of AgNP-based systems for environmental pollutant removal proceeds through well-defined mechanistic pathways that must be explicitly distinguished to interpret performance data. In dark catalytic (reductive) reactions, such as the NaBH_4_-mediated reduction of 4-nitrophenol or azo dyes, AgNPs function as electron-relay platforms: Borohydride ions adsorb onto the Ag surface, donating electrons that lower the activation energy barrier for hydride ion transfer to the substrate. This process follows pseudo-first-order kinetics, with smaller particle sizes (higher surface-to-volume ratio) and electron-rich surfaces (modulated by capping agents) yielding higher apparent rate constants [122–123]. In plasmonic photocatalytic systems, AgNPs and Ag–semiconductor composites harness LSPR via two dominant mechanisms. First, hot-electron injection, where LSPR decay produces energetic electrons that inject across the Schottky barrier into the conduction band of the coupled semiconductor (e.g., TiO_2_, ZnO, or g-C_3_N_4_) within ≈150 fs, generating long-lived charge-separated states [124–125]. Second, near-field electromagnetic enhancement, in which the intensified local electric field around AgNPs boosts electron–hole pair generation in adjacent semiconductor domains [126]. Schottky junction formation at the Ag/semiconductor interface creates an internal electric field that suppresses electron–hole recombination, extending carrier lifetime and enabling band-alignment-dependent redox reactions [127]. Reactive oxygen species, primarily •OH, •O_2_^−^, h^+^, and ^1^O_2_, drive oxidative pollutant mineralisation; their identity and relative contributions must be verified experimentally using EPR spin-trapping (DMPO adducts for •OH/•O_2_^−^) and selective scavenger assays rather than inferred solely from degradation percentages [128–129]. Finally, the apparent quantum efficiency should be reported normalised to incident photon flux to allow meaningful cross-study comparisons and to quantify true photonic utilisation [130]. These mechanistic insights, grounded in ultrafast spectroscopy, EPR, and scavenger studies, reveal why AgNP composites often outperform pristine semiconductors and enable rational design of next-generation environmental catalysts. However, various parameters, such as pH, temperature, catalyst dose, and pollutant concentration, are important in designing nanoparticle-based catalytic applications. Importantly, many high-performance examples cited above involve Ag-containing composites (Ag/TiO_2_, Ag/ZnO, Ag-Fe_3_O_4_, Ag/silica) rather than pristine AgNPs (Table 3). The enhanced performance of these systems should be attributed to the composite architecture (Schottky junction, increased surface area, and synergistic ROS generation) rather than to AgNPs alone.

**Table 3:** Include studies on catalytic/photocatalytic degradation/removal of environmental pollutants leveraging the properties of AgNP and AgNP-nanocomposite.

Catalyst	Targeted pollutant	Reaction conditions	Rate constant (*k*)	Recyclability	Advantages	Limitations	Ref.

Ag and Fe-TiO_2_ nanotubes	polyethylene microplastics (100–150 μm)	UV-C illumination, 25 mg microplastics in 250 mL distilled water, 90 min irradiation for microplastics	NA	NA	simple anodization + SILAR synthesis; bandgap narrowed to 3.07 eV (redshift); Schottky barrier enables efficient e^−^ trapping; 18% microplastic weight loss (best among tested); exceptional *E. coli* disinfection (99.999% after 10 min) via Ag + ROS synergistic effect	higher Ag loadings (0.06 M and 0.09 M) cause agglomeration/Ag_2_O formation and reduced activity; Fe modification is detrimental (6–9% loss); no reusability or Ag leaching data; gravimetric method only (no TOC/COD); UV-C-dependent	[116]
Ag/silica nanocomposite	ciprofloxacin	0.12 g catalyst in 200 mL of 40 ppm CIP solution; dark 30 min; sunlight irradiation, 35–38 °C; optimal pH 6.7; reaction time up to 180 min	1st order model; *k*: 2.18 × 10^−2^ min^−1^ (*R*^2^ = 0.9976)	3 cycles / >82% efficiency retention	green one-pot synthesis (agricultural waste + plant extract); 98% D.E.% + 87% TOC removal; high stability and recyclability; effective under natural sunlight; porous silica support prevents Ag NP agglomeration	anions (HCO_3_^−^ > H_2_PO_4_^−^ > Cl^−^ > SO_4_^2−^ > NO_3_^−^) reduce D.E.% (2–22%); long optimal time (180 min); lab-scale synthetic solutions only (no real wastewater); neutral pH sensitivity	[120]
Ag@APC (AgNPs on petroleum asphaltene-derived porous carbon)	4-nitrophenol, 2,4-dinitrophenol, and 2,4,6-trinitrophenol	40 mg catalyst, 0.5 M NaBH_4_, 0.1 mmol/L 4-NP, 25 °C, aqueous medium	pseudo 1st order (k: 0.3340, 0.2570, and 0.2408 min^−1^)	5 cycles / 100% (complete reduction ≤34 min every cycle)	extremely high activity; no detectable Ag leaching; excellent recyclability; carbon support enhances stability	synthesis requires petroleum asphaltene-derived carbon (source availability), limited to nitrophenols	[131]
AgNPs@cellulose aerogel	tetracycline	visible light irradiation (4 h), aqueous TC solution; up to 95% removal; 50 mg nanocatalyst; 30, 50 and 70 ppm TC	pseudo 1st order (*R*^2^ 0.98 (30 ppm), 0.97 (50 ppm), and 0.96 (70 ppm); 0.679 h^−1^ (30 ppm), 0.642 h^−1^ (50 ppm), and 0.406 h^−1^ (70 ppm)	4 cycles / >85% efficiency retention (maintained over 85% after four cycles)	green synthesis using agricultural waste and natural reductant; synergistic adsorption-photocatalysis; high visible-light efficiency; excellent reusability and structural stability	no detailed dark adsorption *q*_max_ or equilibrium data; efficiency likely decreases at high TC concentrations (>50 ppm) due to site saturation/light penetration; visible-light dependence	[132]
Flavonoid fraction of *Psidium guajava* L. leaves-mediated AgNPs	methyl orange and Coomassie Brilliant Blue G-250	20 mg FAgNPs in 50 mL of 10 mg/L dye solution; dark 30 min then irradiation; MO: solar light (0.015 W/cm^2^, ≈450 nm) for 10 h; CBB G-250: UV light (0.02 W/cm^2^, ≈365 nm) for 6 h; RT	NA, focused on degradation efficiency	NA	rapid eco-friendly one-pot synthesis (<10 min at RT); dual function (strong antimicrobial + photocatalytic); effective under solar/UV irradiation; small uniform particles; hydroxyl-radical mechanism (electron transfer from FAgNPs)	no quantitative *k* or recyclability data; no detailed reaction conditions; no leaching or stability testing reported; long irradiation time; no real wastewater matrix validation	[133]
*Portulaca oleracea* leaf extract-functionalized AgNPs	reactive green 19A, reactive blue 59, reactive red 120, reactive red 141, reactive red 2	dye 10 mg/mL; AgNP 10 mg; sonication 30 min; time 60 min; UV irradiation (1 mW/cm^2^, 365 nm); temp (20–50 °C); pH (4–9); for resuability AgNP 20 mg; RG19A (20 mg/L)	NA, degradation efficiency 100% (RG19A), 85% (RB59), 88% (RR120), 90% (RR141)and 80% (RR2)	5 cycles	one-pot green synthesis; strong visible-light photocatalytic activity for multiple reactive dyes without reductant; dual/multifunctional (photocatalytic, antibacterial and antidiabetic potential)	no quantitative rate constant *k*; no recyclability or leaching data; visible-light dependence	[134]
*Clerodendrum infortunatum* leaf extract; Optimized AgNPs	ternary dye mixture: methylene blue, crystal violet, thioflavin T	natural sunlight irradiation, room temperature (≈25 °C), natural pH (~6.9), catalyst dose 1 mg/mL (10 mL in 100 mL solution), initial dye concentration 1 ppm per dye (1:1:1 mixture)	pseudo 1st order: Mixture – CV 0.0331, MB 0.0316, TT 0.0247 (*R*^2^ > 0.990); Individual: MB 0.0593, CV 0.0461, TT 0.0268 min^−1^	4 cycles / <9–12% loss per dye per cycle	spectroscopic optimisation for stable, high-activity AgNPs (≈20 nm size); excellent visible-light photocatalysis (82.89–96.96% degradation in 110 min); reduced ecotoxicity in treated water (seedling growth similar to control); green synthesis (leaf extract); reusable with minimal loss	slower degradation for TT (≈83% after 80 min) due to structure resistance/low adsorption; light dependence (sunlight-driven, no activity without light); minor activity loss over cycles; no Ag leaching quantification	[135]
*Causonis trifolia* leaf extract-mediated AgNPs	methyl orange and crystal violet	sunlight irradiation, 10 ppm dye; 20 mg catalyst; time 100 min; 30 min dark	MO: 79%, CV: 77% degradation under sunlight; 0.0157 min^−1^ (MO), and 0.0150 min^−1^ (CV)	5 cycles (12% drop for MO and 14% for CV dye)	green synthesis; multifunctional (photocatalytic, dual sensing of Hg^2+^/Fe^3+^, antimicrobial); eco-friendly and sustainable	no quantitative or leaching data; no stability testing reported; limited to sunlight-driven process; limited reaction parameter evaluation	[136]
Amoxicillin-derived AgNPs	cefdinir, Cefditoren, Cefixime, Ceftriaxone sodium, doxycycline	RT, aqueous medium, 0.1 M NaBH_4_, 100 μM antibiotic, 0.10 mg catalyst	NA	5 cycles (high retention: 100% to 91–95% efficiency)	one-pot green synthesis; ultrarapid 100% reduction of multiple antibiotics in 2–5 min; reusable 5 times with maintained/enhanced activity; simple, low-cost, no sophisticated apparatus required; excellent for antibiotic-polluted wastewater	No numerical *k* values reported; no Ag leaching quantification; limited to NaBH_4_-assisted reduction (not photocatalytic); no detailed pH/dose optimisation or real wastewater matrix testing in the study	[137]
Bimetallic Ag/Cu nanoparticles (green-synthesised using *Carica papaya* leaf extract)	chlorpyrifos	≈25 °C, 6 mL NPs, degradation monitoring, 21 days pesticide 50 ppm	NA	NA	green one-pot synthesis; unique star-shaped/tentacle-like morphology; efficient room-temperature degradation of toxic chlorpyrifos to less toxic TCP and DETP; high potential for water purification without UV or extra chemicals	no numerical *k* value; no recyclability or Ag leaching data; long degradation time (days for complete conversion); limited to chlorpyrifos model pollutant	[138]
Ag/TiO_2_ nano-composite (1% Ag doped on TiO_2_)	polyethylene microplastics (particle sizes: 100–125, 125–150, 150–250 μm)	UV irradiation, stirring at 2000 rpm, initial microplastic concentration 100 ppm, catalyst dose 50 mg in 100 mL aqueous medium, pH neutral, RT	NA	NA	simple PAD synthesis; reduced band-gap (3.02 eV) enables visible-light response; excellent degradation (100% mass loss in 90–120 min at 125–150 μm); effective for microplastic removal in drinking water	no numerical *k* value; long irradiation time required (up to 120 min); accuracy of mass-loss method limited (0.001 g precision); no reusability or Ag leaching data; synthetic distilled-water matrix only	[117]
Ag/ZrO_2_ (5 wt % Ag nanoparticles on ZrO_2_)	propene (C_3_H_6_), naphthalene (C_10_H_8_), propane (C_3_H_8_), diesel soot	fixed-bed quartz reactor (propene: 1000 ppm C_3_H_6_ + 6% O_2_ + He, 100 mg cat., 50 mL·min^−1^; naphthalene: 150 ppm + 10% O_2_ + He, 100 mg·cat., 30 mL·min^−1^; soot: loose contact 1:10 cat: Printex-U, 10% O_2_, 100 mL·min^−1^, 10 °C·min^−1^)	NA	NA	synergistic Ag-ZrO_2_ effect (oxygen vacancies + O_x_^−^ species); very low *T*_50_ for unsaturated HC (propene/naphthalene); novel application of Ag/ZrO_2_ for these VOC; small Ag NPs	low activity for saturated propane (*T*_50_ ≈ 480 °C); activity loading-dependent (Ag10Z better for soot/propene but Ag5Z optimal for naphthalene); no kinetic rate constants (*T*_50_/*T*_max_ only); no Ag leaching quantification in reaction	[139]

**3.1.3 Membrane technologies incorporating AgNPs.** The advent of technological advancements aimed at providing safe water for individuals led to the incorporation of AgNPs into membrane technology, enabling sustained Ag release to remove pathogens and biofouling microorganisms in a continuous-flow system [22]. Nanosilver-incorporated aminated polyethersulfone was designed to impart antibacterial properties to membrane technology for water treatment applications [140]. The incorporated AgNPs exhibited enhanced antibacterial activity, resulting in sustained Ag release over 25 days, indicating improved membrane lifespan [140]. Another study assessed water permeability, antifouling performance, salt rejection, and dye removal using a nanofiltration membrane of green AgNPs with polyethersulfone [141]. The membrane having AgNPs (0.75 wt %) demonstrates water permeability (36 L/m^2^·h^−1^·bar^−1^), salt rejection (NaCl (32 to 57%), MgSO_4_ (26 to 67%), and CaCl_2_ (27 to 41%)) and lowest fouling along with high flux recovery. The surface property, high porosity, and antimicrobial action of AgNPs have added to the advancement of membrane technology [141]. A study developed a nanocatalytic membrane to remove dyes (MO, MB, and RhB) and achieve antifouling. Dye degradation of 99% and 90% was achieved after the first and fifth cycles, respectively, indicating the efficiency of the engineered membrane [114]. Another study developed a photocatalytic membrane using AgNPs, graphene oxide, carbon nitride, and mixed cellulose ester as substrate. This nanosystem degrades more than 89% of dye even after the 25th cycle and increases flux (3–12 times) compared to the layer-by-layer approach [142]. A hydrophilic PVC-based nanofiber membrane was developed by adding PEG, chitosan NPs, and AgNPs. PEG provides hydrophilicity to the membrane, and AgNPs provide antibacterial properties. The results demonstrate that adding 0.5% AgNPs kills *Colitinja* and *E. coli* bacteria, while 0.5% chitosan NPs are effective against *E. coli* [143]. A membrane consisting of AgCl and COOH-MWCNT was used for ultrafiltration of water. An increased flux of 150% (520 L·m^2^·h^−1^·bar^−1^) was achieved with water. The membrane exhibited enhanced photocatalytic activity under UV, with a flux of 98.1% and an inhibition of 99% for *E.* C*oli* [144]. A membrane consisting of AgNPs, zirconium-doped silicon (Ag/ZrO_2_-SiO_2_) killed 98.86% and 97.42% of *E. coli* and *S. aureus*, respectively [145]. The membrane exhibited a higher rejection rate of 96.74% and flux of 331.36 L·m^2^·h^−1^ for reactive black KN-B (50 mg/L) at 2 bar (25 °C). In addition, the antifouling effect was confirmed by a flux recovery of 91.18% after three cycles [145]. The significant improvement in membrane technology for safe water is due to AgNPs’ multifunctional properties and colloidal/gel stability. The water treatment approach has undergone a substantial revolution with the emergence of nanotechnology. Furthermore, the application of AgNPs in air and soil pollution must be addressed.

#### 3.2 Air applications

Air pollution poses a significant threat to human health and the environment, necessitating the development of efficient and sustainable air purification technologies. The primary air pollutants include particulate matter (PM), gases (CO, NO_2_, SO_2_), metals (Pb), PAHs, volatile organic compounds (VOCs), and dioxins [146]. Nanosilver has emerged as a promising material for air purification and atmospheric remediation owing to its strong photocatalytic, antimicrobial and adsorbing properties. Nanosilver is often used in air filters and coatings to remove VOCs, PAHs, PM, and other contaminants.

**3.2.1 Air purification and indoor air quality.** Incorporating AgNPs into air filters enhances their ability to remove airborne pollutants, such as particulate matter, aerosols and microorganisms, improving air quality and protecting public health [147]. Graphene oxide AgNPs impregnated on polyacrylonitrile nanofibers were designed for dual-functional PPE filters. The membrane exhibited effective antibacterial activity against *S. aureus* and GFP-expressing *E. coli*. In addition, PM with a size of 2.7 μm was removed, indicating the potential of AgNPs for air filtration technology [147]. Another study used green (*Ficus elastica*) synthesised AgNPs for the adsorption of gases (SO_2_ and NO_2_). The study found that 98% of SO_2_ removal was achieved with pseudo-second-order reaction kinetics [148]. A study utilised AgNP-incorporated cotton fabrics for removing bioaerosols from air conditioners [149]. The results showed a reduction in CFU when the filter was modified with AgNPs, achieving 76% and 96% microbial inhibition and retention, respectively [149]. A study demonstrates that AgNP-coated HEPA filters have antibacterial efficiency against *E. coli* and *S. epidermidis* upon exposure to dust particles. The result showed that with AgNPs the antibacterial effect increases even in the presence of some dust particles [150]. It was reported that particulate respirators lack antibacterial properties, despite being used to filter fine airborne particles. The reported study developed AgNP-incorporated particulate respirators for dual functionality (i.e., antibacterial and filtration). The result confirmed the inhibition of two bacterial strains, *S. aureus* and *P. aeruginosa* [151]*.* The functional nanocomposite filter membrane was developed using AgNPs, TiO_2_, GO and polytetrafluoroethylene (PTFE) to remove PM and for antibacterial activity. The membrane removed 99.25% of PM2.5 and inhibited 98.7% of *E. coli* growth. The membrane showed 95% removal efficiency after five cycles [152]. A recent study developed air filter paper using hardwood pulp and glass fibre, incorporating AgNPs to inhibit the growth of *E. coli* and *S. aureus*, with inhibition zones of 1.52 and 2.04 mm, respectively [153]. AgNPs exhibit antimicrobial properties and reduce foul-smelling pollutants, helping maintain air quality. The AgNPs–cellulose derivative-impregnated PP fibres were used to correct air odour by removing sulfur compounds [154]. The removal of foul-smelling pollutants, including hydrogen sulfide (H_2_S) and ethanethiol (C_2_H_5_SH), was achieved at 95% for H_2_S (50 ppm) and C_2_H_5_SH (25 ppm) [154]. An efficient nanosystem was developed using polyamide-6 electrospun nanofibers attached to AgNPs via hydrogen bonding for antibacterial, antiviral and PM removal [155]. The study reported the removal of PM2.5 (99.99%), a low-pressure drop (31 Pa), and the removal of aerosol pollutants (SO*_x_*, NO*_x_*, methylbenzene, and ʟ-nicotine). The nanosystem inhibits *E. coli*, *S. aureus*, and *Porcine deltacoronavirus* with no significant toxicity. This indicates the functional properties of nanosilver in air filtration technology [155]. An Ag/Cu-doped TiO_2_ nanosystem was developed for photocatalytic removal of air pollutants. This nanosystem is effective against both Gram-positive and Gram-negative bacterial strains when exposed to UV-A [156]. AgNPs incorporated in PVDF nanofiber membranes were synthesised for improved air purification, achieving efficiencies of 99.95–99.97% and a pressure of 137.5 Pa. Additionally, 99.6% inhibition of *S. aureus* and *E. coli* was achieved after exposure to sunlight for two weeks [157]. Chitosan-stabilised AgNPs incorporated in silica hydrogel beads were prepared for an air filter with antibacterial properties. In a bacterial medium, the nanosystem inhibited *S. aureus* and *E. coli*, whereas in an air filter, it inhibited *B. subtilis* [158]. A study developed hybrid nanofibrous membranes consisting of CS/AgNPs/PVA/CA for a highly efficient air filtration system. The results were 99.78% (PM2.5) filtration efficiency, low-pressure drop (61.15 Pa), and enhanced zone of inhibition (100–141%) [159]. A side-by-side nanofiber was designed with ZnO on one and AgNPs on the other side for air pollutant removal application. The nanofiber exhibited filtration efficiency (80%), dye degradation (97% after 140 min), and antibacterial activity [160]. Carbon fibre cloths loaded with AgNPs/TiO_2_ were developed for catalytic removal of NO, a common air pollutant and carcinogen. It was reported that 3.70 wt % AgNPs in the nanocomposite showed the highest removal of NO, with minimum and maximum efficiencies of 80 and 95%, respectively. In addition, the generation of NO_2_ occurred at modest levels [161]. AgNP-incorporated PP membranes selectively removed 97% of particles with a diameter of 0.3 μm, which includes PM1.0 and PM2.5. The membrane exhibited broad-spectrum antibacterial activity, with a maximum inhibition zone [162].

**3.2.2 Removal of VOCs.** Volatile organic compounds are major air pollutants that contribute to smog formation and pose risks to human health. These are ubiquitous air pollutants emitted by various sources, including industrial processes, transportation, and consumer products, and pose significant health and environmental risks. AgNPs exhibited light-induced photocatalytic air purification and pollutant degradation [163]. The AgNPs act as electron sinks, promoting charge separation and reducing electron–hole recombination, enhancing photocatalytic efficiency. AgNPs-modified photocatalysts can effectively degrade various air pollutants under UV or visible light irradiation, including VOCs, nitrogen oxides, and PM [164]. AgNPs can be combined with semiconductor photocatalysts, such as titanium dioxide, caesium oxide, or zinc oxide, to enhance their photocatalytic activity and improve the degradation of air pollutants [165–166]. A review article highlighted the role of Ag–CeO_2_ in catalytic removal of CO, soot, and VOCs via multiple mechanisms [167]. A recent study demonstrated the oxidation of acetone and ozone using an Ag-modified (CeO_2_–Al_2_O_3_) nanocomposite, achieving oxidation rates of 96% and 98%, respectively. Additionally, the reaction selectivity exceeded 97%. The relative humidity enhanced gas-phase photolysis in the presence of UV [168]. Bifunctional zeolite-Ag catalysts (ZSM-5||Ag/γ-Al_2_O_3_) showed 100% conversion of HCHO at 55 °C, where 32% conversion was achieved at ZSM-5||Ag/SiO_2_ [169]. A study explored the potential of diesel particulate matter (DPM) oxidation, a hazardous environmental contaminant [166]. A study examined the possibility of Ag-supported different metal oxides (Al_2_O_3_, TiO_2_, ZnO, and CeO_2_) as a nanocatalyst for DPM. The Ag/Al_2_O_3_ nanocatalyst showed enhanced catalysis and reduced ignition temperature by >50 °C compared to Ag/ZnO [166]. A photodynamic nanoconstruct (Ag/UiO-66) was developed for the adsorption of the VOC styrene. An Ag-dependent nanoconstruct exhibited enhanced adsorption and desorption even after five reusability cycles [166]. A nanocomposite containing AgNPs demonstrated toluene removal. The 80Ag-CeO_2_@CNWs/CF demonstrated enhanced catalysis with 10%, 50%, and 90% conversion rates at 222, 240, and 256 °C, respectively. The nanocatalyst exhibited good stability and water resistance, indicating its long-term stability and reusability [170]. Ethylbenzene is a VOC widely used in various industrial applications. The oxidation of VOC was achieved using an AgNPs/ca nanocatalyst synthesised with different Ag/Ca molar ratios, resulting in 90% conversion at varying temperatures, depending on the ratios, with an increase in relative humidity from 0% to 40% [171]. The air filter comprises nanosilver and TiO_2_ for photocatalytic reduction and antimicrobial activity against VOCs, bacteria, and fungi [172]. The VOC degradation was estimated in a 10 m^3^ area and found to be 91.6%, 80%, 70.1%, and 43% for butanol, acetone, diethyl ether, and benzene removal, respectively, after 55, 100, 120, and 150 min. In addition, 99% of bacteria and fungi were killed after the airflow [172]. A study described AgNPs on graphite carbon nitride (Ag/g-C_3_N_4_) for photocatalytic activity for air purification. This system removed various VOCs (formaldehyde, acetaldehyde, ethylene, benzene, toluene, and *p*-xylene) and killed *E. coli*, *S. aureus*, and *C. albicans* [173]. These studies have highlighted the role of AgNPs as an efficient material for the remediation of air pollutants. The application of AgNP-based nanosystems in soil pollution management requires attention.

#### 3.3 Soil remediation and agricultural applications

Soil contamination by heavy metals, pesticides, and other pollutants poses a significant threat to both agricultural productivity and environmental sustainability. Nanosilver has shown potential for soil remediation and agrarian applications, offering unique properties and functionalities that enable the removal of contaminants from the soil and enhance plant growth.

**3.3.1 Remediation of soil contaminants.** Nanosilver can bind metals in the soil, such as lead, cadmium, and arsenic, reducing their bioavailability and preventing plant uptake [174]. AgNPs promote the removal of organic pollutants, such as pesticides and herbicides, in the soil through catalytic or redox reactions [174–175]. Fipronil is a broad-spectrum insecticide associated with carcinogenic, neurotoxic, and endocrine-disrupting effects that affect humans via plant uptake [176]. A study hypothesized that adding AgNPs to phytoremediation would significantly enhance fipronil removal. Different formulations of AgNPs with *Brassica*, *Ipomoea*, *Camellia*, and *Plantago* reduced fipronil at 95.45%, 90.15%, 63.65%, and 63.48% (in water) and 68.8%, 54.64%, 43.75%, and 30.99% (in soil), respectively. The combined effect of AgNPs produces fipronil metabolites in *Plantago* roots and leaves, while a reduction of the translocation of fipronil was observed. This study has highlighted the potential of AgNP-mediated phytoremediation of insecticides [176]. Pesticides (methyl parathion) and herbicides (pendimethalin and trifluralin) were removed via catalytic reduction using ZnO nanostars doped with Ag and Pd. The results showed that Ag and Pd doping improved catalytic reduction, with Pd-doped nanostars performing better than Ag-doped nanostars [177]. AgNPs were synthesised in a mixture of *Orthosiphon stamineus* leave extract and an ionic liquid using an electrochemical method; they degraded the herbicide 2,4-dichlorophenoxyacetic acid. The herbicide degradation was 65.61% while as per RSM, 97.8% removal was achieved at pH (3.24), catalyst (0.009 g/L) and herbicide (8.15 mg/L) [178]. Activated carbon has good adsorption capacity due to its porous structure. Therefore, AgNPs decorated with activated carbon were synthesised to remove glyphosate. The adsorption efficiency with AgNPs was 149.25 mg/g, compared to 42.92 mg/g for activated carbon only [179]. Additionally, metal ions were removed. The membrane selectively removed Cd and Pb after 40 and 60 min, with adsorption capacities of 625 and 370.37 mg/g at neutral pH [180]. Although this methodology was initially developed for an aqueous environment, it can be optimised for use with future technology on soil samples. Furthermore, antibiotic-resistant bacteria and genes, as well as emerging soil pollutants, significantly impact ecodiversity [181]. A study prepared silver–lignin NPs to inhibit drug-resistant bacterial strains (*S. aureus*, *P. aeruginosa*, *S. epidermidis*, *K. pneumoniae*, and *A. baumannii*) without toxicity on THP-1 cells. The NPs upregulated selectively genes responsible for membrane efflux in *P. aeruginosa* [182]. The AgNPs significantly reduce the metal content of As, Cr, Pb, Mn, and Cu by 75%, 69%, 62%, 86%, and 76%, respectively, in *Zea mays* plants. The AgNP concentration-dependent accumulation reduces metal ion levels and improves plant shoot, root, and vigour index. The AgNPs increased antioxidant activity, carotenoids, chlorophyll a and b, and reduced malondialdehyde in the plants, indicating the potential of nanosilver-mediated plant growth and bioremediation [183]. The other study focused on the stabilisation of Pb ions using nanocomposites (AgNPs–TiO_2_ NPs) in cowpea (*Vigna unguiculata* (L) Walp). The nanocomposite significantly reduced the malondialdehyde content (29%), increased carotenoids (88%), and enhanced antioxidant activity. In addition, 57% of Pb was immobilised, restricting its translocation from soil to shoots [184]. Green (cocoa pod)-synthesised AgNPs were evaluated for the immobilisation of Cd and Pb in the plant (*Moringa oleifera*). The results showed that improved plant physiological and biochemical parameters were obtained compared to the control, indicating that AgNPs mediated plant growth and metal ion removal [185].

**3.3.2 Agricultural applications and ecotoxicological considerations.** Nanosilver is widely used in plant disease management, growth promotion, and soil improvement. AgNP-based formulations can be applied to plant surfaces to control fungal and bacterial diseases, reducing the need for synthetic fungicides and bactericides. AgNPs can disrupt the cell membranes of plant pathogens, inhibit their growth, and prevent disease outbreaks in agricultural fields [186]. AgNPs can be used as seed coatings or foliar sprays to protect plants from diseases and pests, reducing crop losses and improving agricultural productivity. Another study validated the role of foliar AgNPs as phytoremediation agents in removing metal ions. The impact of AgNPs on the growth of *Abelmoschus esculentus* plants grown in soil from a gold mine was evaluated. There was a significant reduction of heavy metal ions and other contaminants by 60% and 44%, respectively [187]. Spraying AgNPs onto plants infected with Tomato mosaic virus and Potato virus Y significantly reduced viral load at 50 ppm. The TEM analysis confirmed the surface attachment of AgNPs to the virus coat, along with the induction of photosynthesis, total soluble protein, peroxidase, and polyphenol oxidase activity [188]. Duckweed (*Lemna minor*)-stabilised AgNPs were synthesised and evaluated regarding insecticidal activity. The green AgNPs exhibited insecticidal activity against *Sitophilus oryzae* (IC_50_ 6.08 µg/mL) and *Tribolium castaneum* (IC_50_ 7.03 µg/mL) along with significant wheat seed germination during one year of storage [189]. This suggests that AgNPs can serve as a protective seed-coating material to promote growth and sustainability. AgNPs have emerged as a promising tool for immobilising heavy metals and facilitating the degradation of organic pollutants in contaminated soils. A study investigated the impact of nanosilver combined with growth-promoting rhizobacteria on plant growth and metabolism in plants irrigated with municipal wastewater. This study confirmed that treating wastewater with nanosilver and rhizobacteria enhanced plant growth and increased the bioremediation potential against toxic metal ions [190]. *Azadirachta indica*-stabilised AgNPs inhibited the growth of phytopathogens with efficiencies of 92%, 89%, and 69% for *Penicillium sp.*, *Fusarium sp.*, and *Aspergillus sp.*, respectively. Exposure to *Ralstonia solanacearum* resulted in cellular damage, bulging, pit formation, and nucleic acid discharge, ranging from 8% to 37%. This indicates that AgNPs can be used as agrochemicals [191]. Rice crop is hampered by bacterial leaf-blight disease caused by *Xanthomonas oryzae pv. oryzae* (Xoo). AgNPs stabilised with *Bacillus cereus* (SZT1) exhibited an inhibition zone of 24.21 ± 1.01 mm against Xoo [192]. Upon modulating the surface chemistry with chitosan, the MIC and MBC were 2.5 and 20 μg/mL, respectively [193]. Green AgNPs can aid in the germination of aged rice seeds. The result showed that priming aged rice seeds with AgNPs enhanced germination efficiency and seedling vigour. ROS and upregulated aquaporins enhance seed germination and starch metabolism [194]. Several studies have highlighted the role of AgNPs in enhancing plant seed germination and promoting plant growth [195]. However, promotion of AgNPs as agrochemicals, seed treatments, or plant growth promoters must be balanced with rigorous ecotoxicological assessment. At elevated concentrations, AgNPs exhibit phytotoxicity, manifesting as inhibited seed germination, reduced root elongation, and chlorophyll degradation in crop species [196]. Sub-lethal concentrations disrupt soil microbial communities, particularly nitrogen-fixing bacteria (*Rhizobium sp*.), nitrifiers (*Nitrosomonas sp.*), and decomposers, which are critical for organic matter cycling [197]. Earthworm and soil invertebrate toxicity has been documented at concentrations exceeding 100 mg/kg soil dry weight [198]. Bioaccumulation of silver in plant tissues and its potential transfer through the food chain raise additional concerns regarding human dietary exposure from AgNP-treated crops [196]. The regulatory status of AgNPs as agrochemicals remains ambiguous in most jurisdictions; neither the US EPA nor the European Chemicals Agency (ECHA) has established maximum residue limits specifically for silver nanoparticles in food or soil [31,199]. Any agronomic application of AgNPs should therefore be preceded by a comprehensive ecotoxicological risk assessment under realistic soil and crop conditions, and the results should be reported alongside efficacy data to ensure a balanced evaluation.

### 4 Environmental monitoring

**Figure 4:**
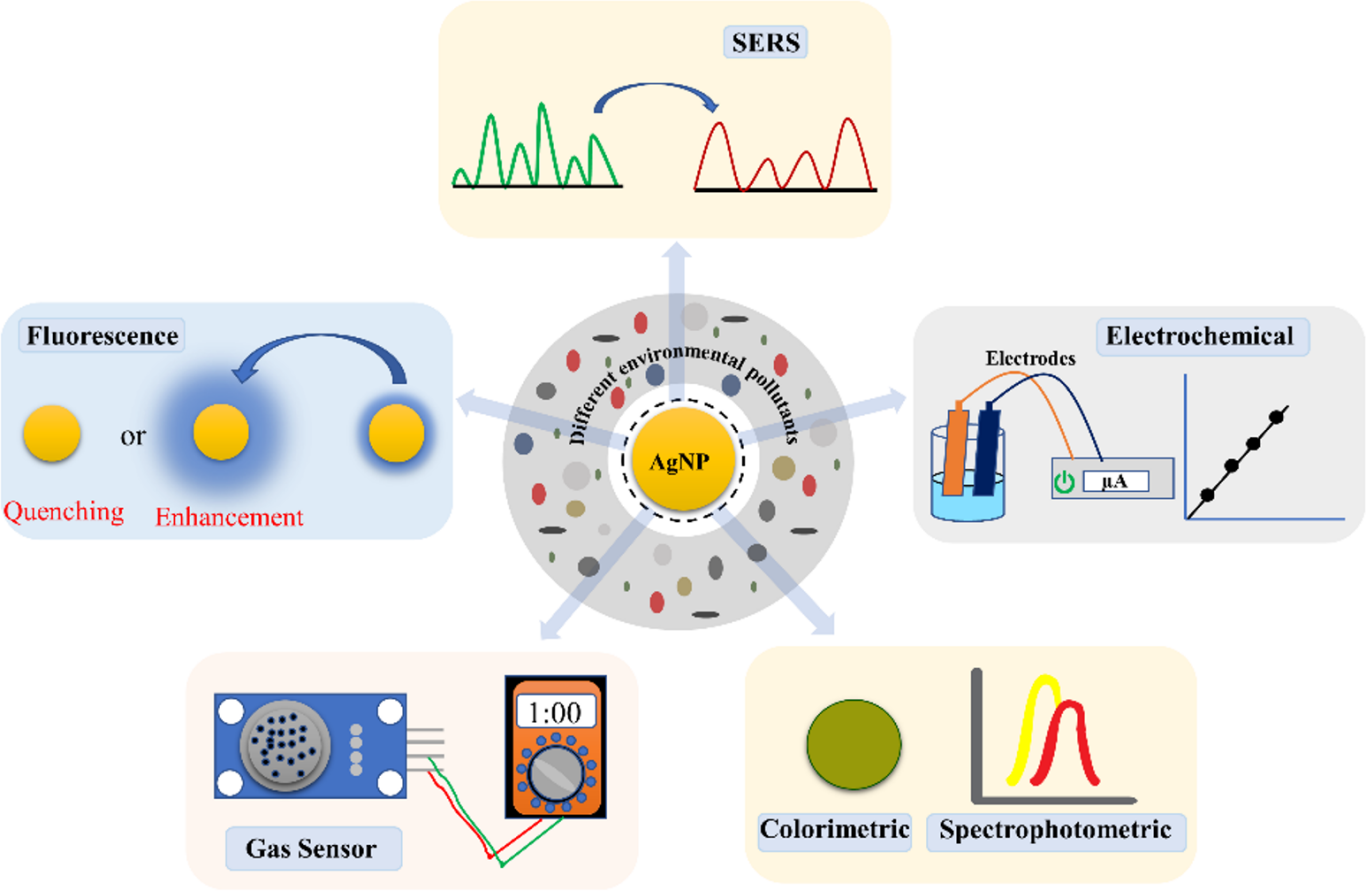
Analytical approaches employing silver nanoparticles in environmental monitoring applications.

Environmental monitoring is crucial for determining pollution levels, identifying contamination sources, and assessing the effectiveness of remediation efforts. Nanosilver, with its unique optical properties, makes it a promising sensing element for environmental monitoring applications, offering high sensitivity, selectivity, and stability for detecting various pollutants and contaminants in water, air, and soil [200]. The strong optical properties of AgNP-based nanosensors enable the monitoring of environmental pollutants, such as metal ions, pesticides, and microplastics [18,201]. The target specificity of nanosilver can be modulated by the NP's physical and chemical properties [200]. The detection methods are based on electrometric, spectrometric, fluorometric, and SERS techniques (Figure 4).

#### 4.1 UV–vis spectrometric/colourimetric

Spectrophotometric sensors developed using AgNPs operate on the principle of LSPR. The LSPR of AgNPs changes in response to interactions with their surroundings. This leads to a visible shift in the colour of NPs. A study using citrate-AgNPs detected eight metal ions depending on their plasmonic shift. A new peak emerged with Zn^2+^, Cu^2+^, Cd^2+^, Fe^2+^, Mn^2+^, and Hg^2+^, whereas Ni^2+^ and Pb^2+^ induced a redshift in SPR [202]. Another study reported size-dependent metal detection using ʟ-carnosine-AgNPs, in which As^3+^, Cr^3+^, Cd^2+^, and Pb^2+^ were detected via a colourimetric/spectrophotometric approach [8]. The LODs for metal detection were 1.2, 0.63, 2.8, and 4.7 μM for Cd^2+^, Pb^2+^, As^3+^, and Cr^3+^, respectively [8]. Also, CTAB-AgNPs were able to detect Hg^2+^ and Cu^2+^ using spectrophotometric methods [9]. In addition to metals, 3-glycidoxypropyltrimethoxysilane-capped AgNPs were used to detect thiram (a fungicide) via LSPR changes. The prepared AgNPs measured thiram in various samples, including river and tap water, fruit and vegetables, and soil, with a LOD of 213 nM [203]. AgNPs functionalized with α-cyclodextrin showed colourimetric detection of chlorpyrifos by a redshift from 410 to 570 nm in SPR. Nanosilver detected chlorpyrifos in fruit and vegetables at concentrations of 4 and 13 ng/mL (LOD and LOQ, respectively) [204]. Casein-functionalized AgNPs were used to detect streptomycin via spectrophotometric measurement at 405 and 520 nm, accompanied by a visual colour change from yellow to orange. The methods showed LODs of, respectively, 98 and 56 nM and linearities of, respectively, 200–650 and 100–700 nM in the tap water and dairy samples [205]. Querecetagetin-AgNPs detected amoxicillin, where a redshift was observed. The method exhibited a LOD of 4.47 μM with a regression coefficient of 0.993 [206]. PVA-AgNPs detected ampicillin in milk and water using a simple colourimetric approach. A redshift from 402 to 550 nm was observed upon adding antibiotics to AgNPs, with LOD and LOQ of 6.2 and 18.8 μM, respectively [12]. The visual detection of mercaptan gases, including methyl mercaptan (CH_3_SH), ethyl mercaptan (C_2_H_5_SH), and butanethiol (C_4_H_5_SH), was performed using (dodecanethiol, oleic acid, and oleyl amine)-functionalized AgNPs immobilized on polyvinylidene fluoride substrates. LODs of 80, 28, and 109 ppb were obtained for CH_3_SH, C_2_H_5_SH, and C_4_H_5_SH, respectively, after 10 min exposure [207]. Another study showed that visual detection of formaldehyde, acetaldehyde, propionaldehyde, glutaric dialdehyde, and hydroxy formaldehyde was achieved using AgNP-decorated paper with LODs of 9 ppb, 3.1 ppm, 3.5 ppm, 23.8 ppb and 71.5 ppb, respectively [208].

#### 4.2 Surface-enhanced Raman spectroscopy

Surface-enhanced Raman spectroscopy (SERS)-based detection is a highly sensitive method for identifying and analysing compounds, including single molecules. It enables the identification and measurement of trace analytes by amplifying the Raman scattering signal of molecules adsorbed onto rough metallic surfaces or nanostructures. SERS-based detection of six metal ions was achieved using pectin-functionalized AgNPs [209]. 4-Mercaptobenzoic acid-functionalized AgNPs were used to detect metal ions, with selective detection of Cu^2+^ at a LOD of 2.5 × 10^–7^ M and linearity over 10^–3^ to 10^–7^ M. In addition, Fe^3+^, Co^2+^, Mn^2+^ and Pb^2+^ were detected, indicating the potential for multimetal detection using a single nanoprobe [210]. Flower-like AgNPs were used to detect pesticides in green tea via SERS. The pesticides, namely acetamiprid, methomyl, and 2,4-dichlorophenoxyacetic acid, were detected with LODs of 1.88 × 10^−4^, 5.58 × 10^−4^, and 4.72 × 10^−3^ µg/mL, respectively. The SERS-dependent monitoring system is highly sensitive and specific [211]. A AgNP/TiO_2_ honeycomb structure was used to monitor micro- and nano-polystyrene plastics in different water sources via SERS. The nanoprobe selectively detected microplastics in water samples from tap, lake, soil, and sea with detection limits of 100, 100, 100, and 250 μg/mL, respectively [212]. Nickel oxide nanosheets decorated with AgNPs served as SERS substrates for detecting various microplastics, including polyethene (PE), polypropylene (PP), and polystyrene (PS). The method exhibited high sensitivity for PS, PE, and PP, with detection limits of 5, 25, and 25 μg/mL, respectively. Notably, PS was detected in water, milk, and liquor with sensitivities of 25, 25, and 50 μg/mL, respectively [213]. An AgNPs/Au@AgNRs sandwich structure was used as a substrate for SERS-based detection of various PFAS, namely, perfluorooctanoic acid (PFOA), perfluorohexanoic acid (PFHxA), and potassium perfluorobutanesulfonate (PPFBS) [214]. The SERS substrate GO@PVP@Ag detected the PAH phenanthrene, with a LOD of 10^−8^ g/L [215]. PAH detection with a LOD of 0.5 nM was achieved using SERS enhancement in Fe_3_O_4_@GO@Ag [216]. Oxytetracycline was detected using a glucose-modified AgNPs SERS sensor. The methods worked perfectly on honey samples with a detection limit of 5–20 ppb [217]. Isepamicin detection was performed using BSA-stabilised AgNPs modified with α-Fe_2_O_3_. The method exhibits a good linear range (20–2000 nmol/L), a LOD of 16.58 nmol/L, and a recovery of 96.29–104.12% [218]. AgNPs decorated SiO_2_ were used to detect VOCs, and their detection limits for toluene, benzene, chloroform, and acetone were 68, 56, 129, and 161 ppm, respectively [219].

#### 4.3 Electrochemical sensors

An electrochemical sensor is a device that converts the chemical response into a signal detectable by modern electrical instruments. The sensors use amperometric, potentiometric, voltammetric, and conductometric measurements [220]. Incorporating AgNPs into electrochemical sensors can be achieved through various strategies, including electrode surface modification, signal amplification, and catalytic enhancement of redox reactions. Folic acid-modified AgNPs were used to detect Hg^2+^ ions using electrochemical methods via cyclic voltammetry (CV) and electrochemical impedance spectroscopy (EIS). Differential pulse voltammetry (DPV) was used to quantitatively estimate Hg^2+^ at 8.43 μM under optimised reaction parameters [221]. Electrochemical detection of the pesticide imidacloprid was achieved using a nanocomposite (Ag@Meso-C/Hematite Ore)-modified GCE. Amperometric and linear sweep voltammetric techniques were employed for quantitative analysis [222]. SDS-stabilised AgNPs were used for electrochemical detection of clothianidin, with a reduction peak at −300 mV vs Ag/AgCl and a LOD of 2.4 nM [223]. A voltammetric sensor for ciprofloxacin was developed using green (*Camellia sinensis*) synthesized AgNPs attached with carbon black. The two linear ranges were 3.1–24.8 µmol/L and 36.9–130.3 µmol/L, and the LOQ was 0.48 µmol/L [224]. Nanosilver deposited on carbon paste detected metronidazole using EIS. Linearity was observed within the range 10^−6^ to 10^−3^ M, along with LOD and LOQ values of 2.06 × 10^−7^ and 6.88 × 10^−7^ M, respectively [225]. The nanosensor (MWCNs-poly(2-aminothiophenol)@AgNPs) exhibited sensitivity in detecting divalent metal ions (Pb^2+^ and Cd^2+^) in food samples. The method showed good linearities (0.5–60 nmol/L and 8–50 nmol/L) and LODs (0.125 and 1.47 nmol/L) for Pb^2+^ and Cd^2+^, respectively [226]. Electrochemical detection of PVC microplastics was performed using a nanocomposite (AgNPs/GO/MWCNT) in both seawater and soft water. The Au-modified electrode showed −0.30 V vs Ag/AgCl with linearity (1–5 mg/mL) and a LOD of 0.79 mg/mL [227]. PFOS detection was performed using MXene-AgNP, with LOD and LOQ of 33 and 99 ppq, respectively, in the linear range of 50 ppb−1.6 ppt [15]. An electrochemical sensor based on carbon-dot-stabilised AgNPs was used to detect PAHs, including anthracene and naphthalene. The sensor exhibits enhanced sensitivity for anthracene (250 nM–1.15 mM) and naphthalene (500 nM–842 μM), with LODs of 112 and 383 nM, respectively [228].

#### 4.4 Fluorometric sensors

Fluorometric sensing based on nanosilver has emerged as a powerful technique for sensitive, selective detection of environmental contaminants. The underlying principles are fluorescence quenching or fluorescence enhancement. For example, PVP-AgNPs detected Hg^2+^ using OPD as a substrate and AgNPs exhibiting nanozyme activity (i.e., enzyme-mimicking catalytic behaviour arising from surface-mediated electron transfer, not from a proteinaceous active site). The method found metal concentration-dependent fluorescence quenching of nanozymes in the 20–2000 nM range and a LOD of 8.9 nM [229]. The four kinds of polymer-templated silver nanoclusters discriminated among seven different metal ions. This sensor had a linear range of 15–800 μM at a pH of 7 [230]. The quantitative estimation of ceftriaxone and cefepime was performed by quenching AgNP fluorescence in a dose-dependent manner. The method works at 1–10 and 0.9–9 µg/mL with LODs of 0.178 and 0.145 µg/mL for ceftriaxone and cefepime, respectively [231]. Oxytetracycline and erythromycin were detected using fluorescence quenching with LODs of 2.714 and 3.306 nM, respectively, using graphene carbon dots modified with Ag as a nanoprobe [232]. The fluorescence-enhancing approach was utilised to develop a fenitrothion sensor using MOF embedded with methionine AgNPs (Meth-AgNPs@Fe-BTC). Under optimised conditions, the obtained linearity and LOD were 2–95 nM and 1.9 nM, respectively [233]. In another study, Zr-MOF embedded AgNPs (AgNPs@PCN-224) were used to detect fenitrothion with a LOD of 0.037 ng/mL and linearity (0.1–500 ng/mL) [234].

#### 4.5 Gas sensors

Gas sensors are electronic devices that measure the presence and concentration of specific gases in the air or other environments. It converts the chemical interaction between the sensor material and the target gas into an electrical signal. This signal enables the identification and quantification of the gas. AgNPs can be used for gas-sensing monitoring of various gases, either alone or as components of composite materials. AgNPs and AuNP-modified ZnO were used to detect ammonia gas at room temperature (RT). A surface-modified nanosensor exhibited enhanced detection with LODs of 0.495 and 0.405 ppm for AuZn and AgZn, respectively [235]. In another study, NO_2_ detection was achieved using AgNP-doped ZnO nanorods. The response rate increased to 434.3% at 250 °C and 1 ppm, while the detection limit reached 1 ppb [236]. A resistive gas sensor was developed using a nanocomposite (ZIF-8/MWCNT/AgNPs) to detect VOCs (methanol, ethanol, acetone, acetonitrile, and *n*-hexane) at RT [237]. A chemiresistive H_2_S sensor was formulated using AgNPs-decorated graphene, achieving detection below 6 ppb after 6 min [238]. TiO_2_/AgNPs heteronanostructures detected CO_2_ [239]. Silver nanoparticle-based sensors can monitor environmental conditions in real time, providing valuable data for informed decision-making and risk assessment. Table 4 represents the overall sensing ability of AgNPs and their composites for environmental pollutant monitoring reported in the literature.

**Table 4:** Including AgNP and their nanocomposite for environmental pollutant monitoring.

Nanosensor	Pollutant	LOD and linear range	Matrix	Validation	Advantages	Limitations	Ref.

MXene-AgNPs composite	PFOS and other long-chain PFAS	LOD 33 ppq, LOQ 99 ppq; linear range 50 ppq–1.6 ppt (for PFOS; *R*^2^ = 0.998)	aqueous solutions; industrial wastewater	reproducibility: *n* = 3–5 independent electrodes; selectivity vs short-chain PFAS	synergistic AgNP electrostatic + MXene F−F binding; ultrasensitive ppq detection in 5 min; low-cost, portable, no interferences from common ions/surfactants; direct impedimetric; stable	optimal only for long-chain PFAS (low response to short-chain); requires sample dilution for high concentrations; minor matrix effects in complex wastewater	[15]
Citrate + PVP-stabilized AgNPs	Al^3+^	40.5 nM and 0.1–1000 nM	real water samples (tap, pond, river, mineral)	recovery: 96–113%; repeatability RSD <8%; intermediate precision RSD <12%; high selectivity vs other metals	simple colourimetric (no complex functionalization); highly selective; successfully applied to real samples; lower LOD than many Ag/AuNP sensors	pH-sensitive (instability at pH <3; 70% absorption drop in 24 h at pH 3); minor Ni^2+^ interference at high ratios (100:1)	[240]
*Persimmon leaf* extract-mediated AgNPs	Hg^2+^	0.1 ppb (visual); 4.415 ppb (calculated); 0.1–100,000 ppb (*R*^2^ = 0.9913)	aqueous medium; spiked tap water	recovery >99% in spiked tap water (vs AAS reference); high selectivity (no interference from selected metals)	eco-friendly, rapid (10 min synthesis), ultrasensitive, stable (1 month), portable, no instruments needed; dual use (colourimetric + antibacterial)	requires pH optimisation (optimal pH 7); potential aggregation in extreme pH	[241]
Sodium alginate-functionalized AgNPs	dimethoate	30 ppb; 0.05–2 ppm (*R*^2^ = 0.99)	actual water samples	satisfactory recoveries in real water; high selectivity	rapid visual colourimetric (via aggregation); wide linear range; simple, stable probe	limited reusability data; matrix effects in complex waters not fully quantified beyond selectivity	[242]
Chitosan + citrate-capped AgNPs	paraquat	10 μM and 10–100 μM (*R*^2^ = 0.98)	siked real samples (soil: farm/lake/black/red/brick; food: tomato/distilled water/tap water)	RSD: repeatability 5%, reproducibility 4%, interference 2%, real-sample analysis 3.5%; recovery in spiked soil 74.2–95.2%	highly selective; rapid; portable/flexible paper platform; low-cost/on-site colourimetric; sustainable; RSD within limits; naked-eye detection	reaction time ≈9 min; tested only on spiked/pre-treated matrices; no long-term shelf-life/stability data; relies on specific electrostatic aggregation	[243]
β-CD-SH-mediated AgNPs	Nile blue, Nile red, fluconazole, carbendazim, benz(a)anthracene, bisphenol A	10^−9^ M (Nile blue and Nile red); 10^−6^ M (fluconazole, carbendazim, benz(a)anthracene, bisphenol A); linear 10^−6^ to 10^−9^ M	aqueous/water environment	RSD <10% (9.8% intra-film, 6.3% batch-to-batch at 10^−7^ M Nile blue); spiked recovery 75%; excellent uniformity vs control	Ultra-rapid (40 s) one-step assembly + analyte trapping in hotspots; excellent reproducibility; integrates substrate formation and capture; demonstrated real environmental application (Nile blue release from PMMA nanoplastics)	requires organic phase (hexane/ethanol) for assembly; lower sensitivity for hydrophilic analytes; recovery <100% (75%); excessive β-CD-SH reduces signal	[244]
Ag/SWNTs@CuBTC-MOFs composite	Hg^2+^ (pH 5), Ni^2+^ (pH 7), Fe^3+^ (pH 10)	LOD and LOQ: Hg^2+^ (1.39 and 1 nM), Ni^2+^ (2.6 and 1 nM) and Fe^3+^ (3.03 and 1 nM); sensitivity 7.84 µA/nM Hg^2+^, 2.21 µA/nM Ni^2+^, 0.866 µA/nM Fe^3+^	aqueous buffer (acetate, phosphate and glycine-NaOH buffer)	high selectivity (no response to Cd^2+^, Cr^2+^, Pb^2+^, Zn^2+^ at 1 µM); LOD below US EPA max limits; pH-dependent detection; stability retained 93.5% after 60 days	multianalyte detection with pH-tunable selectivity/sensitivity; high electrochemical activity (e.g., 7.84 µA/nM for Hg^2+^); no ion pre-accumulation needed; suitable for water monitoring	pH-specific; complex multistep synthesis/activation (120 °C/24 h); no real-matrix spiking/recovery data (only buffer); long-term stability drops to 93.5% after 60 days	[245]
AgNPs-PYRO nanosensor	fouling ions (Ba^2+^, Sr^2+^, Mg^2+^, Ca^2+^, Fe)	detectable 0.5–10 ppm	aqueous water; real oil-field scale relevance implied	colour change; plasmon band decrease at ≈400–420 nm; zeta potential shift on Ba^2+^ addition; DLS size increase; FE-SEM aggregation	simple, rapid (15 min incubation), low-cost colourimetric + UV–vis detection; distinguishes divalent/trivalent ions by colour; stable colloid; aggregation-based mechanism	ppm-range detection; higher sensitivity mainly for Ba^2+^; no real oil-field brine spiking/recovery data; aggregation method may suffer interference in a complex system	[246]
Chemically induced AgNPs (portable intelligent colourimetric sensor platform)	H_2_S, SO_2_, and NO_2_ gases	H_2_S: LOD 12.8 ppb (linear 0.5–5 ppm); SO_2_: LOD 131.8 ppb (linear 0.5–20 ppm); NO_2_: LOD 23.1 ppb (linear 0.5–10 ppm); 5 min exposure	gaseous phase (airbox, 25 °C, 50% RH); also chicken spoilage headspace	excellent selectivity; PCA/HCA clustering (96.56% variance); humidity resistance (20–90% RH, no loss of activity); real meat freshness validation	portable/smartphone-integrated visual detection; ultrasensitive ppb LODs; rapid/cumulative (5 min); humidity-resistant; distinguishes gases by unique colour patterns; no instrumentation needed; 30 day stability	response relies on aggregation/anti-aggregation (morphology/size change); primarily lab airbox tested (limited complex real-air matrix data); indirect meat monitoring via headspace gases only	[247]
Ag nanoarrays	polystyrene nanoplastics (PS NPs; 130 nm, 180 nm, 230 nm sizes)	10 µg/mL (all); linear 10–100 µg/mL and 100–1250 µg/mL (DI water)	DI water; real environmental water (river, rainwater, tap); also tested with interferents (NaCl, CaCl_2_, SDS, glucose at 0.1 wt %) and multiplastic mixtures	RSD = 6.8% after ≥30 reuse cycles; excellent stability and reusability; size-matching confirmed by SEM + 3D-FDTD simulations; RSD 6.8%	size-matching optimises electromagnetic hot-spots for maximum SERS enhancement per PS size; reusable and stable; works in complex real waters; portable/rapid	requires custom AgNAs fabrication for each PS size; LOD in µg/mL range; toluene cleaning required for reuse; performance drops if PS size mismatches AgNAs spacing/height	[248]
NiO/AgNP nanowell	polystyrene (PS), polyethylene (PE), polypropylene (PP)	PS: 5 µg/mL (standard), 25 µg/mL (water); PE/PP: 25 µg/mL (standard), 50 µg/mL (milk/liquor); linear 100–2500 µg/mL (*R*^2^ 0.9684–0.9893)	standard aqueous; real beverages (drinking water, milk, liquor); interferent-tested (NaCl, MgSO_4_, protein, sugar)	excellent uniformity (RSD 5.94–10.61% at 20 points); 30 day stability (14% intensity drop); spiked recoveries 86–110.7% (RSD 4.44–13.44%); multiplastic distinction; coffee-ring + hydrophobicity validated by SEM	nanowell + LSPR + charge-transfer (NiO-PS) for hotspot capture; portable Raman; rapid (dry droplet); works in complex beverages; quantitative linearity	µg/mL LOD in µg/mL; droplet drying step; minor Ag oxidation over time; matrix-dependent LOD increase in milk/liquor	[213]
Ag nanocubes @black Si nano-assemblies	polystyrene nanoplastic beads (500 nm)	SiNTs: LOD 57.92 µg/mL (linear 25–500 µg/mL); SiNPs: LOD 21.43 µg/mL (linear 25–500 µg/mL)	aqueous solutions	reproducibility: RSD ≈8.3% (SiNTs) / ≈1% (SiNPs) at 1347 and 1600 cm^−1^ (13 spots); CV probe EF 4.2 × 10^9^ (SiNTs) / 1.9 × 10^8^ (SiNPs)	simple/low-cost fabrication (DRIE + eco-friendly AgNCs); 3D geometry for hot-spots and high EF; size/shape control for PS capture; comparable/superior LOD vs literature	LOD in µg/mL; specific to 500 nm PS (size/geometry matching needed); NaCl aggregation required; no real-matrix (e.g., beverages) validation	[249]
AgNP-decorated electrospun nanofibrous nylon-6	GenX (hexafluoropropylene oxide dimer acid / HFPO-DA)	1 ppb and 100 ppm to 100 ppt (*R*^2^ = 0.99 at 751 cm^−1^; *R*^2^ = 0.99 at 813 cm^−1^)	water (lab-prepared GenX solutions	selectivity demonstrated (unique Raman peaks distinguish GenX from PFOS, PFOA, PFBA); high enhancement factor EF = 1.3 × 10^7^	ultrasensitive (1 ppb), rapid (minutes), cost-effective, non-destructive; scalable for field wipes/swabs; size-specific AgNP optimisation yields strong SERS	AgNP size critical (≈60 nm optimal); no recovery/RSD/reusability data reported; no real matrix assessment	[250]
β-CD/Ag-decorated MOF	PFAS, primarily PFOA; also PFNA, PFOS, PFBA, PFHxA	40 ng/L (1.723 × 10^−10^ M) for PFOA; linear range 10^−7^–10^−10^ M at 721 cm^−1^; full detection 10^−2^–10^−10^M	aqueous solutions	reproducibility: 20 spots on 5 MOFs; SERS mapping at 721 cm^−1^; stability	direct SERS of PFAS; microfluidic + waveguide extends light-analyte interaction; rapid; LOD below EPA 70 ng/L; PCA enables differentiation	linearity/repeatability needs optimization; manual peak discrimination is difficult for similar-chain PFAS (relies on PCA); requires flat liquid surface control	[251]
AuNS@Ag nanostar 2D array	PAHs (pyrene and phenanthrene)	pyrene: LOD 10^−8^ M (linear 10^−3^–10^−8^ M at 1412.76 cm^−1^, *R*^2^ = 0.9792); Phenanthrene: LOD 0.5 × 10^−8^ M (linear 10^−5^–0.5 × 10^−8^ M at 1049.38 cm^−1^, *R*^2^ = 0.9707)	aqueous solutions	reproducibility: 5 random points per spectrum; uniformity mapping; 2-week air stability; EF = 5.28 ×10^7^ (R6G at 1372.75 cm^−1^)	bimetallic synergy + nanostar “lightning rod” + 2D array plasmonic resonance for ultrahigh EF; portable; stable/uniform solid substrate; rapid (30 s); overcomes PAH hydrophobicity via silane modification	signal decreases slightly with storage (day 1–6); linearity requires log-scale fit; optimal only after Ag shell tuning (16 µL AgNO_3_); hydrophobic modification step required	[252]
Nanosilver-silicon coupling substrate	phenanthrene (Phe), fluoranthene (Flt), fluorene (Flu), naphthalene (Nap)	Phe (0.542 µg/g; 2–55 µg/g), Flt (0.342 µg/g; 2.7–60 µg/g), Flu (0.541 µg/g; 2.3–53 µg/g) and Nap (5.132 µg/g; L 3.065 µg/g)	oily sludge	uniformity: RSD 2.8% (Phe), 1.08% (Flt), 1.41% (Flu), 5.44% (Nap); stability (7 batches, RSD <6.29%); PLS external validation (MREP 0.0580–0.0713); SNR enhancement vs no substrate	simple/rapid substrate prep; high uniformity/stability/sensitivity (trace 2 µg/g); methanol extraction + hot-spot formation; PLS/SiPLS-VIP enables accurate quantification in complex matrix	higher LOD for Nap (5.132 µg/g); requires spectral preprocessing and variable selection for optimal PLS; methanol extraction step; baseline/fluorescence interference without preprocessing	[253]
AgNP-functionalized GO/ionic liquid composite	malathion	1–200 nM range (*R*^2^ = 0.9987)	aqueous solutions	good consistency; interference (ethanol/isopropanol/methanol 10× conc., RSD 1.48%); reproducibility (RSD 1.05%, multiple sensors); repeatability (RSD 2.04%); long-term stability	synergistic GO (selectivity via oxygen groups), AgNPs (electron transport), BMIMPF₆ (ionic conductivity/stability); PVC/o-NPOE framework boosts mechanical/electrochemical reliability; high sensitivity/selectivity; low-cost LIG fabrication	quadratic (not linear) response; framework materials required for optimal stability; tested primarily in controlled aqueous/acetone (limited real-matrix validation shown)	[254]
Core–shell Ag@MIPs-CaF_2_	3,6-dichloro-2-methoxybenzoic acid (dicamba)	0.16 µM (3σ/k); linear range 1.0–50.0 µM (*R* = 0.9994)	aqueous solutions; spiked real food samples	recoveries 85.4–103.5% (RSD <4.1%, *n* = 3) vs HPLC; selectivity (minimal response to benzoic acid, 4-chlorobenzoic acid, quinclorac, acetamiprid, etc., and ions K^+^/Ca^2+^/Na^+^ etc. at 50 μM)	MEF (7.1× enhancement via optimal Ag–CaF_2_ distance + spectral overlap at 450 nm); ratiometric self-calibration; MIPs specificity; visual detection under UV; simple synthesis; high imprinting factor	optimal performance requires a precise TEOS (30 µL) and template: monomer (3:10) ratio; tested mainly in spiked food extracts; quadratic/linear response limited to 50 μM upper range	[255]
rGO/AgNPs hybrid nanomaterial	paracetamol (PA) and caffeine (CF)	PA (0.0152 µM, 0.1–10 µM or 0.2–15 µM single); CF (0.7146 µM, 0.1–10 µM or 0.1–13 µM single); simultaneous: 0.1–10 µM for both	0.1 M PBS (pH 6); commercial pharmaceutical tablets	recoveries 84.76–114.53% (RSD <4.34%, *n* = 3) vs labelled values; selectivity (no interference from 1 mM glucose/AA/OA); reproducibility (RSD 0.30–4.34%, 3 electrodes); stability/reusability (≤2% decay after 6 months storage)	eco-friendly green synthesis; synergistic rGO (high surface/conductivity) + AgNPs (electrocatalysis, anti-aggregation); low LODs, wide range, excellent peak separation; simple low-cost CPE; real-tablet applicability; high stability	optimal performance requires precise 12% loading; primarily validated in buffer/tablet extracts; scan-rate/pH optimisation needed; irreversible process limits some kinetic studies	[256]
Carbon dots/AgNPs	ketotifen (KTF)	0.981 µg·mL^−1^; 3–40 µg·mL^−1^ (*R*^2^ = 0.9996)	eye drops and artificial aqueous humor	recoveries 96.97–104.33% (RSD <2.6%, *n* = 3); ICH-validated (accuracy, precision, robustness, selectivity vs excipients); no interference from common ophthalmic components	green one-step microwave synthesis; instantaneous response; simple visual/colourimetric readout; high selectivity via aggregation; sustainable; real-sample applicability without complex preparation	single-analyte focus; optimal performance requires pH 4.0 buffer and precise CDs/AgNPs volume; tested only in eye drops/aqueous humor	[257]
Ag@SiNWs	ceftriaxone	plasma: LOD 94 µM, linear 1–1000 µM (*R*^2^ = 0.9361); microdialysate: LOD 1.4 µM, range 2.5–1000 µM (*R*^2^ = 0.9427)	spiked fresh plasma; microdialysate	concentration-dependent SERS spectra; linear/Langmuir fits; stability; selectivity vs matrix; protein precipitation validated by peak enhancement	high SERS enhancement via Ag hotspots on SiNWs; microdialysate reduces protein interference; fast real-time readout suitable for TDM/POCT; stable substrates; molecular specificity	protein precipitation required for plasma; higher LOD in plasma vs microdialysate; Langmuir in microdialysate; individual batch fabrication	[258]
Au-Ag@Au fibre SPR sensor	enrofloxacin (Enro), ciprofloxacin (Cip)	Enro: 0.97 ng·mL^−1^; Cip: 0.70 ng·mL^−1^; linear range 0–50 ng·mL^−1^	milk samples	recoveries 96.64–115.32% (Enro), 96.18–121.06% (Cip) (RSD <3%); stability (no decay after 15 days PBS or 1 h 1% H_2_O_2_); FOM 14.92–20.23	high sensitivity/portability; simultaneous multiantibiotic detection; oxidation-resistant Au shell protects Ag core; simple, scalable fabrication; real-milk applicability; label-free real-time monitoring	requires PDA/Ab functionalization and sequential sandwich assay; milk recovery variability (up to 121%); linear range capped at 50 ng·mL^−1^; sulfite critical for uniform Au shell	[259]

#### 4.6. Analytical validation and metrological considerations

A rigorous analytical validation is a prerequisite for translating AgNP-based sensing performance from laboratory settings to practical implementation. LOD and linear range were almost universally reported, whereas comprehensive metrological characterization remains inconsistent across the literature. Accuracy, expressed as percentage recovery in spiked real environmental samples, typically ranges from 95 to 120%. For example, the GO-BMIMPF_6_-AgNPs@PVC-o-NPOE impedimetric sensor for malathion achieved recoveries of 95–105% in spiked aqueous matrices with excellent agreement to reference methods [254]. Similarly, the Ag@MIPs-CaF_2_ core–shell platform for pesticides delivered 84–104% recovery in spiked water and food samples, while the rGO/AgNPs hybrid nanomaterial for the detection of pharmaceutical residues reported 84–114% recovery in real samples [255–256]. Precision, expressed as relative standard deviation (RSD), is reported in only a minority of studies, but it consistently falls within the 1–6.9% range in well-characterised platforms. The GO-BMIMPF_6_-AgNPs@PVC-*o*-NPOE sensor showed intra-day RSD of 1.05–2.04% and inter-day RSD of 1.48% across replicate measurements, while the Ag@MIPs-CaF_2_ substrate exhibited <4.1% signal uniformity [254]. Matrix effects remain a major challenge in complex environmental samples (e.g., humic acids, competing ions, surfactants), often leading to nonspecific LSPR shifts or electrode fouling. Successful mitigation strategies documented in the literature include standard-addition protocols, matrix-matched calibration, and controlled sample dilution, which enabled the high recoveries cited above. Sensor-to-sensor and batch-to-batch reproducibility is rarely systematically evaluated; most studies report only intra-electrode RSD, underestimating the variability introduced during AgNP synthesis. Storage stability and shelf-life are infrequently quantified; the GO-BMIMPF_6_-AgNPs@PVC-*o*-NPOE platform retained functionality for 30 days under refrigeration with only 15.61% drift, and the Ag@MIPs-CaF_2_ composite maintained 95.5% of its initial intensity after 28 days at room temperature [254–255]. Long-term stability data (>3 months) remains limited for most platforms. These meteorological gaps hinder regulatory acceptance and field deployment. Table 4 provides a study-by-study assessment of each sensor’s performance against common environmental pollutants, including advantages and disadvantages.

### 5 AgNPs versus alternative nanomaterials: a critical comparison

The position of AgNPs within the broader nanomaterials landscape requires direct, quantitative comparison with alternative materials across each application domain. In photocatalysis, pristine anatase TiO_2_ (bandgap 3.2 eV) is active exclusively under UV irradiation (λ < 385 nm). Ag/TiO_2_ composites reduce the bandgap to 2.49–2.84 eV through LSPR-driven hot-electron injection and plasmonic near-field enhancement, extending activity into the visible range and increasing apparent rate constants two- to fivefold (or higher) under solar or visible-light irradiation. For example, TiO_2_/Ag (9.3%) achieved *k*_app_ = 2.451 × 10^−2^ min^−1^ for MB degradation under visible light (>400 nm), compared with much lower values for pristine TiO_2_ [260]. ZnO prepared by facile precipitation (ZnO-400) already matches or exceeds TiO_2_ (P25) in specific photoactivity for methyl orange and phenol degradation under UV irradiation [261]. Photodeposition of Ag further boosts performance, and the resulting Ag/ZnO-400-R composite achieved significantly higher degradation rates for methyl orange. It can be reused for at least 18 cycles without loss of activity, demonstrating the electron trapping role of metallic Ag nanoparticles [261]. Graphitic carbon nitride (g-C_3_N_4_, bandgap ≈ 2.7 eV) is an intrinsic visible-light photocatalyst whose activity is further enhanced by Ag decoration, achieving a 2.5-fold higher degradation rate (*k*_app_ = 0.01576 min^−1^ vs 0.00626 min^−1^) and 80% vs 48% removal of malachite green after 100 min under UV irradiation compared with pristine g-C_3_N_4_. The improvement arises from metallic Ag nanoparticles (≈6 nm) acting as electron traps, thereby suppressing electron–hole recombination [262]. In sensing applications, AuNPs and AgNPs are the principal LSPR-active materials. Spherical AgNPs exhibit 5–10-fold higher LSPR extinction coefficients than AuNPs of equivalent size, with LSPR peaks at 390–500 nm (optimally overlapping with the spectral response of sensitive photodetectors), making them preferable for colourimetric and SERS platforms. Direct head-to-head comparison of AgNP vs AuNP-based cobalt-porphyrin-DNA genosensors shows nearly identical performance: Both achieve LODs of 5.0 × 10^−18^ M (AgNP) and 3.8 × 10^−18^ M (AuNP) with the same linear range (5 × 10^−17^–1 × 10^−16^ M), detecting only ≈30 and ≈23 target DNA molecules, respectively. The redox signal is dominated by the cobalt porphyrin (not the NP), and both exhibit excellent selectivity [263]. The AgNP system offers superior DNA economy (lower probe loading) and lower material cost while maintaining equivalent signal-off behaviour, whereas AuNPs offer an easy-to-set-up system for laboratory testing [263]. A study confirmed that AgNPs outperform AuNPs as reporter labels in electrochemical point-of-care immunoassays [264]. Critically, AgNP detection requires only a one-step potential sequence in the presence of chloride (no corrosive HCl addition), enabling true user-friendly operation, whereas AuNP assays require an additional acid step, which is incompatible with point-of-care formats. AgNP–antibody conjugates furthermore exhibit excellent storage stability (≥4 weeks) and sharper stripping peaks at lower potentials [264]. For electrochemical sensing, graphene and carbon-nanotube platforms provide higher electroactive surface areas and faster heterogeneous electron-transfer kinetics than AgNP-modified electrodes, but lack the dual catalytic and optical functionality that renders AgNPs uniquely versatile [265–266]. However, designing AgNP-based adsorbent materials for water pollution control applications is challenging. For a more robust critical appraisal, the reported maximum removal/adsorption capacities must be contextualised against both conventional benchmarks and emerging high-performance materials. Conventionally, activated carbon is considered a better material for achieving 50% removal of methyl orange (2 mg/L, pH 7), whereas the addition of AgNPs significantly improved the adsorption rate to 72.5%, indicating improved efficiency and recyclability up to ten cycles [267]. Ding et al. reported that nanosilver-loaded activated carbon derived from waste rice noodles achieved a maximum adsorption capacity (*q*_max_) of 97.07 mg·g^−1^ for Cr(VI), markedly higher than the 65.43 mg·g^−1^ obtained with the unloaded activated carbon under identical conditions (pH 2, room temperature) [268]. Biochar is conventionally used as an adsorbent. For instance, the AgNP/biochar nanocomposite and biochar showed strong adsorption, removing 98.18 ± 1.46% and 87.72 ± 1.58% of Cr(VI) at 10 ppm/10 mL after 240 min, respectively. In addition, the nanocomposite removed 90.10 ± 1.31% of nigrosine at 10 mg/L after 60 min, while the study lacked a biochar assessment [269]. Similarly, for graphene oxide, Vicente-Martínez et al. showed that GO functionalized with AgNPs (GO@AgNPs) achieved 100% phosphate removal instantaneously at a lower adsorbent dose, compared to only 75% after 9 min with a higher dose of pristine GO [270]. For metal–organic frameworks, Mirzajani et al. found that AgNPs encapsulated in ZIF-8 MOF delivered *q*_max_ values of 76.34 mg·g^−1^ for Cd^2+^ and 79.36 mg·g^−1^ for Cu^2+^ in real water matrices, providing added antibacterial functionality absent in pristine MOFs [101]. Direct comparisons for zeolites remain limited, but AgNP decoration of related composites has shown improvements in heavy-metal uptake analogous to those observed for zeolites. Overall, AgNP integration consistently enhances adsorption capacity, kinetics, and reusability, while providing secondary benefits (e.g., antimicrobial or magnetic recovery).

In antimicrobial applications, AgNPs demonstrate clear superiority over AuNPs and other common nanomaterials such as TiO_2_ and ZnO through direct mechanistic and quantitative comparisons. AgNPs exert broad-spectrum bactericidal effects primarily via Ag^+^ ion release, ROS generation, membrane disruption, and interaction with thiol groups in proteins and DNA, achieving MIC values in the range of 1.4–5.6 μg/mL and MBC values of 2.8–5.6 μg/mL against both Gram-positive and Gram-negative strains [271]. Aggregated data across hundreds of studies confirm that AgNPs exhibit MIC of 0.11–1200 μg/mL (MBC of 0.22–1500 μg/mL) and demonstrate broader efficacy against Gram-negative bacteria (64% of studies) due to electrostatic attraction and thinner peptidoglycan layers [272]. In comparison, AgNPs produce equivalent antibacterial zones at 4.86 μg/mL, whereas AuNPs require 197 μg/mL to inhibit *S. aureus* [273]. AgNPs exhibit distinct potency and oxidative-stress mechanisms compared with CuNPs, ZnONPs, and TiO_2_NPs. Direct IC_50_-based growth-inhibition assays across *E. coli*, *B. cereus*, and *S. epidermidis* reveal AgNPs as the most potent against *E. coli* (IC_50_ = 7.84 mg/L), outperforming ZnONPs (176.10 mg/L) and CuNPs (180.80 mg/L), while TiO_2_-NPs dominate in *B. cereus* (50.30 mg/L) and CuNPs in *S. epidermidis* (112 mg/L) [274]. Strain- and NP-dependent alterations in antioxidant defence directly correlate with antibacterial efficacy, positioning AgNPs as highly effective via rapid ROS overload in Gram-negative species, whereas ZnO/TiO_2_NPs require higher concentrations and light-dependent mechanisms for comparable activity [274]. Overall, AgNPs combine the lowest effective concentrations, light-independent action, and broadest spectrum, positioning them as the preferred antimicrobial nanomaterial when rapid, non-photocatalytic bacterial inactivation is required [271–273]. In summary, AgNPs excel in niche multifunctional roles requiring simultaneous optical, catalytic, antimicrobial, or plasmonic properties, while TiO_2_/ZnO dominate UV photocatalysis, AuNPs offer stability in sensing, graphene/CNT provide superior electrochemistry, Fe_3_O_4_ enables magnetic recovery, and MOFs/biochar deliver high-capacity adsorption. Integration of different functional aspects using different materials to form an Ag nanocomposite may provide additional advantages over conventional benchmark systems.

### 6 Challenges

**Figure 5:**
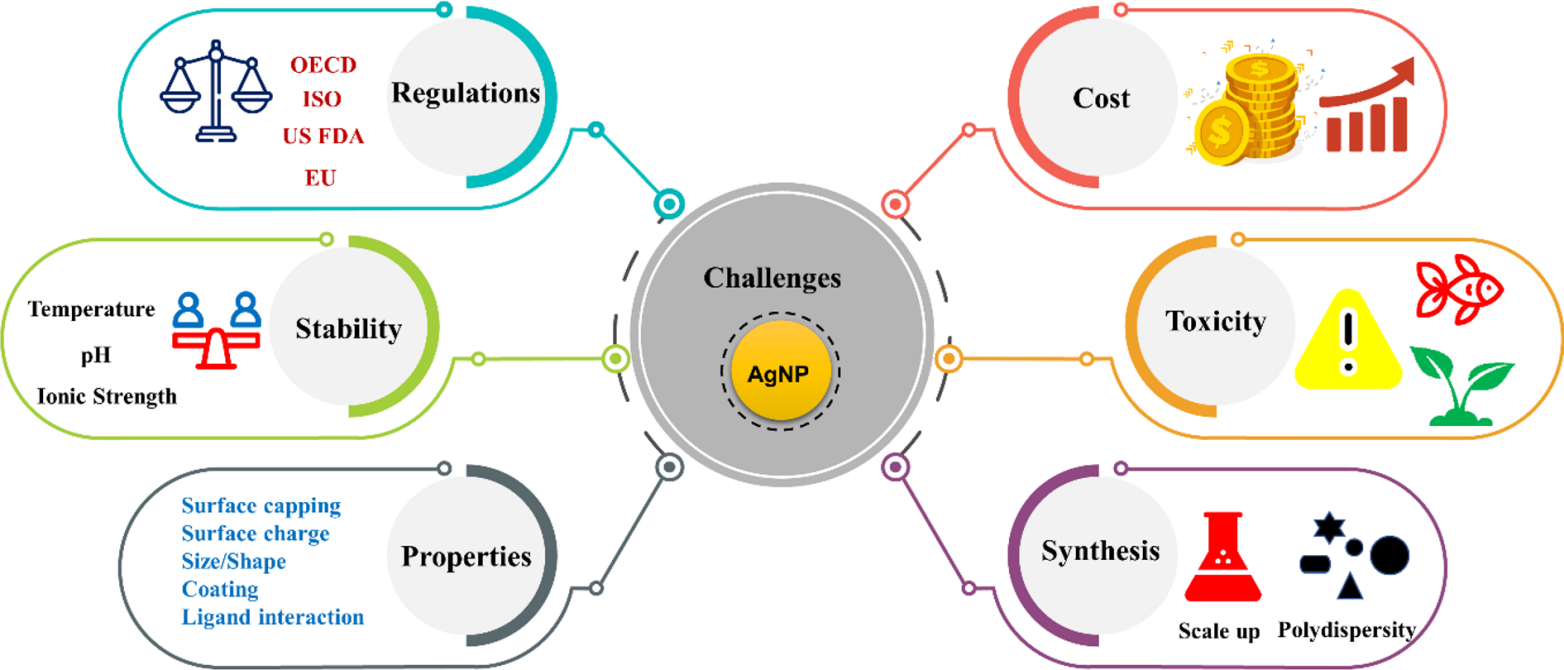
Challenges and limitations associated with silver nanoparticle-mediated environmental applications.

Nanosilver has advanced environmental science applications. Despite their widespread advantages, specific challenges exist which can limit the use of AgNPs or demand a more sustainable material (Figure 5).

#### 6.1 Standardisation, reproducibility, and inter-laboratory challenges

Standardisation and reproducibility represent pervasive, underaddressed challenges across all AgNP-based environmental applications. Batch-to-batch variability in AgNP synthesis arises from subtle differences in reducing agent concentration, mixing rate, temperature, and nucleation kinetics, producing size distributions, zeta potentials, and LSPR peak positions that can vary significantly between preparations nominally following the same protocol. This variability directly propagates into functional performance, causing irreproducibility in sensing LODs, catalytic rate constants, and antimicrobial inhibition zones. Among all the challenges, the synthesis of desired AgNPs for particular applications is the primary concern; for example, small NPs are more efficient antibacterial agents and can be used in filtration membranes than larger ones [47]. Thus, achieving a small size is paramount for antibacterial applications in water, air, and soil treatment. Similarly, optically active AgNPs can be used to monitor and remediate environmental contaminants. The optical properties can be controlled via various synthetic methods. While environmentally preferable, green synthesis methods using plant extracts or microorganisms often suffer from poor reproducibility and limited control over nanoparticle characteristics. Using chemical reducing agents presents concerns about toxicity, while physical methods are energy-intensive and expensive. Additionally, maintaining colloidal stability without aggregation over time requires careful surface functionalization, which can alter the particles’ environmental behaviour and effectiveness [63]. Other challenges are reproducibility, synthesis scale-up with controlled properties, and cost-effectiveness.

Furthermore, regulating AgNPs represents unique challenges due to their novel properties, diverse applications, and potential environmental and health impacts. One of the primary challenges is the scarcity of comprehensive data on the toxicity and environmental fate of silver nanoparticles, which hinders thorough risk assessments and the establishment of safe exposure limits [275]. Analytical techniques that accurately measure and characterise AgNPs in complex environmental matrices are needed to monitor their presence and behaviour [275]. Another challenge is the rapid pace of innovation in nanotechnology, which drives continuous development of new silver nanoparticle formulations and applications. Several regulatory agencies have formulated guidelines to provide direction for the use of nanomaterials. International organisations, such as the OECD, formulate guidelines for the physicochemical properties of nanoparticles (test guides 125, 124, and 318) and safety (test guides 412, 413, and 39) [276]. Other agencies include ISO, EU, and US FDA programs for managing nanomaterial characterisation and safety perspectives [276]. Nationally, the Department of Science & Technology (DST) and the Department of Biotechnology (DBT) have developed guidelines for the safe handling of nanomaterials.

Pursuing sustainable synthesis methods for AgNPs is crucial for reducing environmental impact and promoting green chemistry principles. Green synthesis approaches employing plant extracts and bacterial and fungal filtrates offer eco-friendly options for nanoparticle synthesis [277]. The extracts comprise various bioactive compounds, including polyphenols, flavonoids, and terpenoids, which provide dual functionality to nanoparticles [278]. Biosynthesised approaches contain natural ingredients that are less harmful to people and the environment [279]. The morphology and size of the produced nanoparticles are influenced by the extract concentration, reaction temperature, pH, and silver salt concentration [280–281]. Optimising the reaction conditions is crucial for controlling the size, shape, and stability of the synthesised AgNPs. Green synthesis methods offer several advantages, including reduced toxicity, lower energy consumption, and the use of renewable resources, making them attractive for large-scale production of AgNPs [282]. Recovery and recycling of AgNPs from environmental matrices are essential for promoting resource efficiency and minimising ecological contamination [283]. Effective separation techniques, such as filtration, centrifugation, and magnetic separation, are required to isolate AgNPs from complex environmental samples. A study suggested that AgNPs can be adsorbed on microplastics and thus removed [99]. Monitoring AgNPs in ecological samples via cloud point extraction (CPE) combined with ICP-MS was achieved [284]. Eco-design principles can be applied to minimise the environmental impacts of silver nanoparticle-containing products throughout their life cycle [285]. The manufacturing industry needs to adopt a circular approach, where products are designed intentionally for multiple lifecycles [286]. Additionally, optimising AgNP dosage and delivery methods can minimise their release into the environment and reduce potential exposure risks. Implementing proper waste management practices, including collection, treatment, and recycling, is crucial for preventing the release of AgNPs into the environment and minimising their potential impacts on ecosystems and human health. Further research is needed to develop effective and scalable technologies for removing AgNPs from wastewater and other environmental matrices.

#### 6.2 Environmental fate, transformation, and toxicity of AgNPs

The environmental fate of AgNPs following release is essential for accurate risk assessment and for contextualising the performance data reported throughout this review. The utilisation of AgNPs for environmental applications has raised concerns about their potential ecological release and subsequent ecotoxicological impact through interaction with various organisms [287]. Once released into environmental compartments via wastewater treatment plant (WWTP) effluent, sludge land application, or atmospheric deposition from AgNP-containing consumer products, AgNPs undergo a suite of abiotic and biotic transformation processes that profoundly alter their bioavailability and toxicity. Environmental fate and behaviour of AgNPs depend on multiple factors, including particle size, surface charge, coating, and environmental conditions such as pH, ionic strength, and organic matter content [288–289].

Sulfidation is the reaction of dissolved Ag^+^ with sulfide (S^2−^) under anoxic conditions to form Ag_2_S, which represents the dominant long-term silver sink in anaerobic sediments and WWTP sludge and substantially reduces bioavailability [288–289]. At the same time, chlorination precipitation of AgCl in chlorinated drinking water and saline environments lowers the activity of free Ag^+^ ions. Photochemical reduction converts Ag^+^ back to Ag^0^ nanoparticles under UV–vis irradiation in the presence of organic electron donors, creating photochemical cycling and complexation with dissolved natural organic matter, which can both stabilise AgNPs against aggregation and compete with biological ligands for Ag^+^ [288–289]. The relative rates of these processes depend on the receiving environment (freshwater, saltwater, soil, or sediment) and on AgNP surface chemistry. For example, citrate-capped AgNPs were evaluated for stability in various water samples over 180 min, during which tap water was found to significantly induce aggregation in nanoparticles [290]. Exposure to AgNPs can lead to enhanced ROS production and increased mitochondrial permeability, potentially resulting in mitochondrial DNA damage, cellular apoptosis, and pro-inflammatory responses [291]. AgNPs release Ag^+^ at rates governed by particle size, surface area, dissolved oxygen concentration, pH, and coating identity. In antimicrobial studies, both Ag^+^- and particle-dependent mechanisms (direct membrane contact, ROS generation at the particle surface) contribute to toxicity, and their relative importance varies with organism type and exposure medium. In photocatalytic and nanozyme applications, surface-bound electron transfer is primarily particle-dependent, while dissolution contributes to Fenton-type chemistry. In colourimetric sensing, LSPR shifts are particle-dependent, while certain electrochemical responses exploit Ag^+^ activity. Failure to decouple these contributions through appropriate controls such as dialysis membranes, ion-exchange pre-treatment, or Ag^+^ scavengers leads to mechanistic misattribution and complicates cross-study comparison.

Nanosilver bioaccumulation and biomagnification in food chains pose additional concerns, as they can lead to higher exposure levels in top predators and potentially disrupt ecosystem functioning. Studies have shown that AgNPs can accumulate in various organisms, including bacteria, algae, invertebrates, and fish, with potential consequences for their health and reproduction [32]. The accumulation of AgNPs in aquatic organisms can disrupt their physiological processes, leading to inflammation, oxidative stress, and tissue damage [292]. The antimicrobial properties of AgNPs can have unintended consequences for microbial communities, which play essential roles in nutrient cycling, decomposition, and bioremediation. Exposure to AgNPs can alter the composition and function of microbial communities, potentially disrupting ecosystem processes and affecting the health of higher trophic levels [293]. Nanosilver at low concentrations exhibited toxic effects on zooplankton, which are mediated by the gut microbiota. In contrast, at high concentrations, mortality resulted from the combined impact of the extinct gut microbiota and accumulated toxicity [294]. Introducing AgNPs into natural brackish waters resulted in distinct changes in bacterial community composition and structure, as well as reduced richness and diversity; anoxic conditions may attenuate these effects [295]. Some studies have shown adverse effects of AgNPs on freshwater organisms, ranging from producers to secondary consumers, with evidence suggesting that at least part of the nanoparticle toxicity is due to oxidative stress [296]. Even at low concentrations, nanosilver can harm the bacterial community in the human gut, as evidenced by reduced gas production and changes in fatty acid methyl ester profiles [297]. AgNPs can negatively affect nitrogen-fixing, nitrifying, and denitrifying microbes in vitro, potentially disrupting nitrogen cycling in ecosystems [197].

### 7 Future perspectives and emerging trends

Nanosilver have garnered substantial attention in recent years due to its unique physicochemical properties and diverse applications across various fields, including medicine, agriculture, and environmental science [298–299]. As research and development efforts continue to expand, several promising future perspectives and emerging trends are expected to shape the trajectory of silver nanoparticle technology. Combining AgNPs with other materials to create hybrid or composite materials is an emerging trend. These hybrid materials can exhibit properties and functionalities superior to those of their components, enabling the development of advanced materials with tailored characteristics for specific applications [300]. For instance, incorporating AgNPs into polymer matrices can impart antimicrobial properties to the resulting composite material, making it suitable for medical devices, packaging, and textiles [301–302]. Combining AgNPs with other nanomaterials, such as carbon nanotubes or graphene, can create synergistic effects and enhance their catalytic, electronic, and mechanical properties [303]. These composite materials can be used in energy storage, sensing, and environmental remediation applications. Combining AgNPs with other cutting-edge technologies, such as 3D printing, microfluidics, and artificial intelligence, is an emerging trend with tremendous innovation potential [304]. Integrating AgNPs into 3D printing processes enables the fabrication of customised devices and structures with antimicrobial properties for medical implants, tissue engineering scaffolds, and environmental sensors [305]. Combining AgNPs with microfluidic devices can create highly sensitive and selective sensors for detecting environmental contaminants, pathogens, and biomarkers [306]. Furthermore, the use of artificial intelligence and machine learning algorithms can optimise the synthesis, characterisation, and application of silver nanoparticles, thereby accelerating the discovery of novel materials and applications [307]. Looking ahead, the field of environmental applications of AgNPs is poised for continued growth and innovation, driven by ongoing research and technological advancements. Further research is needed to understand the long-term environmental impacts of AgNPs fully and to develop strategies to mitigate potential risks. Advances in nanotechnology and materials science will lead to the development of novel silver nanoparticle-based materials with enhanced properties and functionalities for environmental applications. The use of AgNPs in ecological remediation, such as water and soil treatment, is expected to increase as more effective and sustainable technologies are developed. Moreover, the development of nanosensors, which enable in situ, real-time tracking of pollutants, is anticipated to grow rapidly in the coming years. Integrating AgNPs with other nanomaterials and technologies, such as nanocomposites and nanodevices, will create new opportunities for addressing complex environmental challenges.

## Conclusion

This comprehensive review has examined the multifaceted environmental applications of AgNPs across various domains. Our analysis reveals that AgNPs have emerged as versatile tools for environmental remediation and protection due to their unique physicochemical properties. The synthesis methods have evolved significantly, with green synthesis approaches gaining prominence for their reduced environmental impact. AgNPs have demonstrated exceptional capabilities in eliminating pathogens, removing heavy metals, and catalysing the degradation of persistent pollutants in water treatment. Their incorporation into membrane technologies has addressed longstanding challenges such as biofouling. Similarly, AgNPs have proven effective in air purification against airborne contaminants, VOCs, and odour-causing compounds. The agricultural applications of AgNPs offer promising alternatives to conventional pesticides while enhancing plant disease resistance. Furthermore, AgNP-based sensing technologies have demonstrated significant advances in sensitivity, selectivity, and real-time detection capabilities at the laboratory scale. However, widespread field deployment remains constrained by long-term stability limitations, matrix interference in complex real samples, potential ecotoxicological risks of released silver, batch-to-batch synthesis variability, and the absence of a coherent regulatory framework governing AgNP use in environmental applications. The limited application of AgNPs has been hindered by their ecotoxicity, challenges in synthesis, and regulatory concerns. The principles of green chemistry, safe-by-design approaches, and the recycling and reuse of materials provide a framework for overcoming the challenges posed by AgNPs. Furthermore, combining AgNPs with emerging technologies such as artificial intelligence and advanced materials science offers unprecedented opportunities to develop innovative environmental solutions. Ultimately, interdisciplinary collaboration among materials scientists, environmental engineers, ecotoxicologists, and regulatory experts is essential to advancing this field comprehensively.

## Data Availability

Data sharing is not applicable as no new data was generated or analyzed in this study.
